# Long‐Term Stability of Bacterial Extracellular Vesicles Stored at Different Temperatures

**DOI:** 10.1002/jex2.70143

**Published:** 2026-05-25

**Authors:** Anagha Rama Varma, Frank Borris, Parnika Kant, Kaitlyn Sadtler, Parinaz Fathi

**Affiliations:** ^1^ NanoEngineering and MicroPhysiological Systems Section (NEMPSS), National Institute of Biomedical Imaging and Bioengineering (NIBIB) National Institutes of Health (NIH) Bethesda Maryland USA; ^2^ Section on Immunoengineering, National Institute of Biomedical Imaging and Bioengineering (NIBIB) National Institutes of Health (NIH) Bethesda Maryland USA; ^3^ Biomedical Engineering and Technology Acceleration Center National Institute of Biomedical Imaging and Bioengineering Bethesda Maryland USA

**Keywords:** bacteria, extracellular vesicles, gram negative, gram positive, membrane vesicles, storage

## Abstract

Extracellular vesicle (EV) research has revealed EV involvement in a variety of biological processes, with potential applications in both fundamental research and therapeutic development. EVs of bacterial origin have been implicated in host‐immune interactions in the development of a variety of diseases. Despite differences in the membranes of bacterial and mammalian cells, past studies on EV storage conditions have focused on mammalian EVs. In this work, we evaluated the effects of storage temperature on the properties of EVs derived from gram‐positive (*Bifidobacterium longum*) and gram‐negative (*Escherichia coli*) bacteria and stored for a period of 8 weeks. It was hypothesized that storage over time would impact the integrity of the EV samples, particularly at higher temperatures. *B. longum* EV identity was confirmed by western blotting for lipoteichoic acid, GroEL, and RecA, while *E. coli* EV identity was confirmed by western blotting for lipopolysaccharide, GroEL, and Flagellin. Using nanoparticle tracking analysis (NTA), we observed no effect of storage on *E. coli* EV size or concentration but found that storage at 4°C for 4–8 weeks led to increases in *B. longum* EV size. No significant changes in particle size or concentration after storage were observed with microfluidic resistive pulse sensing (MRPS). An apparent increase in macrophage activation by the EVs was observed after 4 weeks of storage, followed by an apparent decrease in macrophage activation after 8 weeks of storage. Although EV protein concentration was stable with storage, the intensity and definition of some protein bands in gel electrophoresis appeared to decrease over the storage period. Overall, even short‐term storage of bacterial EVs led to changes in EV functionality, and size measurements alone were not necessarily indicative of EV stability.

## Graphical abstract

Extracellular vesicle (EV) storage can affect their physical and functional properties. In this work, we evaluated the effects of storage temperature and duration on EVs from a gram‐positive and a gram‐negative bacterial strain. We found that particle size alone does not necessarily capture storage‐induced changes in EV functionality.

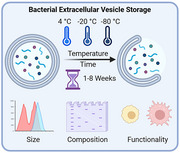

## 1 Introduction

Extracellular vesicles (EVs) are increasingly of interest in a variety of disciplines and applications (Gelibter et al. 2022). Previous studies have amassed evidence that storage conditions affect the integrity of EV samples (Jeyaram and Jay 2017). Many studies have attempted to ascertain the extent of these impacts (Gelibter et al. 2022). Factors such as storage temperature (Lőrincz et al. 2014; Schulz et al. 2020), storage buffer (Gelibter et al. 2022; Kawai‐Harada et al. 2023), length of storage (Gelibter et al. 2022; Lőrincz et al. 2014), and repeated freeze‐thaw cycles (Gelibter et al. 2022; Cheng et al. 2019) can all lead to changes in the stored EV sample, including effects on EV structure and functionality. Accordingly, the International Society of Extracellular Vesicles (ISEV) has set forth guidelines for the proper storage of EVs, noting that storage after EV isolation should be limited, but acknowledging that long‐term storage has often been carried out at −80°C, and that certain types of mammalian EVs have been successfully stored at 4°C and −20°C long‐term (Welsh et al. 2024).

However, the ISEV guidelines note that bacterial EVs are not yet well‐characterized by studies, and that, given their biological variety, it is difficult to issue standards for their use and storage with the same confidence as those issued for eukaryotic EVs (Welsh et al. 2024). Though ISEV states that many of the rules applying to eukaryotic EVs may be applied to bacterial EVs, the guidelines do not give any particular information regarding storage conditions (Welsh et al. 2024). Many of the existing studies in literature examining the effect of storage conditions on EVs focus on mammalian EVs. Furthermore, a recent meta‐analysis found that less than half of the studies on bacterial EVs reported storage temperature and only 5.6% report the storage duration (De Langhe et al. 2024). Bacterial EVs also have relevance to a myriad of topics, including the development of various diseases (Chronopoulos and Kalluri 2020), microbiota‐host interactions (Ñahui Palomino et al. 2021), and therapeutic applications (Jiang et al. 2022; Guo et al. 2021; Hosseini‐Giv et al. 2022). As such, the dearth of storage experiments focusing on the long‐term integrity of bacterial EVs and the lack of standardization in bacterial EV studies must be addressed. Additionally, since gram‐positive bacteria and gram‐negative bacteria produce EVs in different ways (Schorey et al. 2021; Jan 2017; Briaud and Carroll 2020), care must be taken to examine the effects of storage on EVs produced by both types of bacteria.

In order to determine the extent of the impact of storage temperature on the integrity of bacterial EVs, we have performed storage experiments on EVs isolated from a strain of gram‐positive bacteria (*Bifidobacterium longum*) as well as from a strain of gram‐negative bacteria (*Escherichia coli*). It was hypothesized that longer storage time and higher storage temperature would result in decreased sample fidelity. The EVs were subjected to storage at 4°C, −20°C, and −80°C, and their integrity was assessed over the course of 8 weeks. Changes in EV physical properties (size, concentration, and appearance) as well as functional properties (macrophage activation and protein content) were evaluated throughout this time.

## 2 Materials and Methods

### 2.1 Materials

*B. longum* (ATCC 15707), *E. coli* (ATCC 700926), and RAW264.7 cells (ATCC TIB‐71) were purchased from ATCC. Reinforced Clostridial Medium (Hardy Diagnostics C8721) was used for culture of *B. longum*, and Tryptic Soy Broth (Sigma 1054590500) was used for culture of *E. coli*. Dulbecco's Modified Eagle Medium with L‐glutamine (DMEM, Quality Biological 112‐014‐101) was used for RAW264.7 macrophage culture. ExoBacteria OMV isolation kits (EXOBAC100A‐1) were purchased from System Biosciences. Materials for gel electrophoresis, including protein ladder (1610374), 2‐Mercaptoethanol (1610710), Tris/Tricine/SDS Running Buffer (1610744), Silver Stain Plus Kit (1610449), and 8%–16% Mini‐PROTEAN TGX Precast Protein Gels (4561103), were purchased from BioRad. For western blotting, Precision Plus Protein WesternC Blotting Standards (1610376), 10× Tris/Glycine Buffer (1610734), Thick Blot Filter Paper, Precut (1703932), 10× Tris Buffered Saline (1706435), EveryBlot Blocking Buffer (12010020), StrepTactin‐HRP conjugate (1610380), Anti‐LPS antibody (4329‐5004), and Goat Anti‐Mouse IgG [H/L] antibody (STAR117P) were purchased from BioRad. Additionally, 0.45 µm PVDF Transfer Membranes (88585), SYPRO Ruby Protein Blot Stain (S11791), SuperSignal West Pico PLUS Chemiluminescent Substrate (34580), anti‐Flagellin antibody (MA5‐28968), Goat Anti‐Rabbit IgG [H = L] (A16096), and B‐PER Bacterial Protein Extraction Reagent (78248) were purchased from ThermoFisher Scientific. Anti‐GroEL antibody (G6532), Methanol (179957), Acetic Acid (A6283), and Tween‐20 (P9416) were purchased from MilliporeSigma. Anti‐Rad51 antibody (NBP2‐20058) and Anti‐LTA antibody (MA1‐7402) were purchased from Fisher Scientific. The Griess assay reagent systems were purchased from Promega (G2930). Pierce BCA Protein Assay Kits (Thermo Scientific 23227) were used for protein concentration quantification. For particle size and concentration measurements, EVs Membrane Dye (488ex/505em) was purchased from Alpha Nano Tech. Ultrapure Water (Invitrogen 10977‐015) and phosphate buffered saline without Ca or Mg (Quality Biological 114‐058‐101) were used after additional filtration through 0.22 µm PES membrane filtration flasks (CellPro V50022). Nano‐W stain (14702) was purchased from Ted Pella, and 300 mesh carbon‐coated copper transmission electron microscopy grids (CF300‐Cu‐50) were purchased from Electron Microscopy Sciences.

### 2.2 Methods

Summarized methods are provided below, and any applicable additional details are available in the Supplemental Information.

### 2.3 Bacterial Culture

*B. longum* was cultured as the gram‐positive strain of bacteria, while *E. coli* was cultured as the gram‐negative strain of bacteria. Each was cultured using a protocol optimized for the individual strain. Bacterial culture was performed in a shaking incubator. For anaerobic culture of *B. longum*, bacteria were cultured in a 2.5‐L AnaeroJar (Hardy Diagnostics AG025A) using AnaeroGen Anaerobic Gas Generators (Hardy Diagnostics AN25US) in the presence of an anaerobic indicator (Hardy Diagnostics BR55) and without shaking. *B. longum* was cultured using Reinforced Clostridial Medium and *E. coli* was cultured using Tryptic Soy Broth.

Three independent replicates were used for each experiment. The replicates were made independent by staggering the bacterial inoculations to avoid having multiple inoculations of each strain of bacteria taking place on the same day. This was done to account for any minor variations that could occur from one inoculation day to another.

### 2.4 EV Isolation and Storage

EV isolation was performed according to the manufacturer instructions for the Exobacteria OMV Isolation Kit. The kit uses the principle of ion‐exchange chromatography to isolate EVs. A gravity column is first prepared by adding a binding resin provided by the kit and equilibrating with a binding buffer. Following this, the bacterial media from the culture is added to the column and incubated for 30 min at 4°C with rotation on a rack. Once the incubation is finished, the column is opened to allow the media to flow through, then rinsed with the binding buffer. Finally, an elution buffer is used to collect the captured EVs from the column. Although this kit is specified for use in isolating vesicles from gram‐negative bacteria, its use for vesicle isolation from gram‐positive bacteria has also been demonstrated (Naskar et al. 2022, ExoBacteriaTM OMV Isolation Kit (for *E. Coli* and Other Gram‐Negative Bacteria), n.d.), and we have observed good particle yields from use of this kit on *B. longum*‐conditioned medium. Approximately 1.5 mL of EV sample was obtained from each isolation. Samples were kept cool on ice and measurements were collected immediately using fresh sample, before the remaining sample was aliquoted into 0.65‐mL plastic centrifuge tubes for storage.

### 2.5 OD600 Measurements

OD600 measurements were collected using 100 µL bacterial suspension in wells of 96‐well plates. Bacterial medium was used as the blank. Additional OD600 measurements were collected in cuvette format on a ThermoFisher Genesys UV‐Visible Spectrophotometer. For cuvette measurements, 1 mL of bacterial sample was used from each tube and returned to the sample after OD600 measurements were taken. Wellplate OD600 measurements were collected for *n* = 12 samples across four experiments. Cuvette format OD600 measurements were collected for *n* = 9 samples across three experiments. Average OD600 measurements for each sample were calculated using the measurements taken from each tube, with three tubes combined per sample.

### 2.6 Nanoparticle Tracking Analysis (NTA)

For fluorescence‐based measurements, EVs Membrane Dye reagents were prewarmed at 37°C for 10 min. Staining solution was prepared by mixing tracker dye with amplifying reagent at a ratio of 1:4 and mixing thoroughly. The solution was incubated again at 37°C for 5 min. Five microliters of staining solution was added to 20 uL of EVs and mixed thoroughly. Stained EVs were incubated for 30 additional minutes at 37°C before measurements were captured. A 1:100 dilution of EV sample was prepared in 0.22 µm filtered 1× PBS for reading on the NTA. The protocol parameters for the 488 nm laser excitation included a sensitivity of 75 and shutter of 150.

For scattering‐based measurements, a 1:100 dilution of EV sample in 0.22 µm filtered 1× PBS was prepared for reading on a ParticleMetrix Zetaview Quatt Nanoparticle Tracking Analyzer. The protocol parameters for the 405 nm laser excitation included a sensitivity of 80 and shutter of 100, and the protocol parameters for the 488 nm laser excitation included a sensitivity of 70 and shutter of 32.

### 2.7 Microfluidic Resistive Pulse Sensing

A well‐documented limitation of characterizing EVs with NTA, is in the nature of the technique itself, as light scattering techniques tend to bias toward larger particles with high refractive indices (Filipe et al. 2010). This can lead to an overestimation of particle size and an underestimation of particle concentration in EV measurements. Microfluidic resistive pulse sensing can circumvent these limitations in EV samples because it records particle size based on electrical spikes, as vesicles are flowed through a nanoconstriction. For this assay, running buffer was comprised of 1% (v/v) Tween 20 in PBS. Incorporation of a surfactant is important for MRPS to enable particles to flow through the microfluidic channels within the measurement cartridges (Cimorelli et al. 2021). Running buffer was filtered with a 0.2 µm PES filter (WHA67802502, Millipore Sigma) and stored refrigerated for up to 2 weeks. Sample dilution buffer was prepared by filtering the running buffer through a 0.02 µm PES filter (WHA68091002, Millipore Sigma). Sample dilution buffer was freshly filtered for each day. A 1:10 dilution of the EV sample was prepared using the sample dilution buffer. Sample size and concentration were measured on a Spectradyne NCS1, with measurement collection stopped once an average percentage error of 2.2%–2.3% was obtained. This percentage error threshold was selected because it represented a low error value and was also achievable within a short measurement timeframe. C‐400 Spectradyne cartridges were used, which measure particles with diameters between 65 and 400 nm.

### 2.8 Macrophage Exposure to EVs

RAW264.7 macrophages were grown in DMEM supplemented with 10% FBS and 1% penicillin‐streptomycin. One hundred microliters of macrophage suspension (80 × 10^3^ cells/mL) was plated in each well of tissue culture‐treated 96‐well plates. The cells were incubated at 37°C in a 5% CO_2_ environment and allowed to grow for 24 h before medium was replaced with treatment medium. Each treatment condition was done in duplicate on each plate. Treatment medium was prepared by diluting EVs in complete culture medium for final concentrations of 12.5%, 6.25%, and 3.125% (v/v). EV elution buffer at the same final concentrations was used as the vehicle control for macrophage treatment. Regular complete culture medium was used for the untreated control conditions, and untreated controls were included on each plate. The treated plates were incubated for 24 h before medium was collected and stored at −20°C until the Griess assays could be performed.

### 2.9 Griess Assay

Culture medium samples were thawed on ice prior to use in Griess assays. The assays were performed following manufacturer instructions for the Promega Griess Assay Kit. Absorbance at 535 nm was read using a BioTek Synergy H1 and converted to nitric oxide concentration using a standard curve generated for each plate. The nitric oxide concentration detected in the untreated controls on each plate were subtracted from the nitric oxide concentration in the treatment groups on that same plate in order to account for plate‐to‐plate variability.

### 2.10 Bicinchoninic Acid (BCA) Assay

EVs were diluted in 1× PBS for the following final concentrations: 25%, 12.5%, 6.125%, and 3.125%. The Pierce BCA Assay Kit was used, and manufacturer instructions were followed. 562 nm absorbance measurements were collected using a BioTek Synergy H1. To calculate the protein concentration of the undiluted samples, the resulting protein concentration from the 25% samples were multiplied by 4.

### 2.11 Bacterial Protein Isolation

Bacterial cells were pelleted by centrifugation at 3200 × *g* for 20 min. The cell pellets were submerged with B‐PER bacterial protein extract reagent at a concentration of 4 mL per gram of cells. The solution was incubated at room temperature for 15 min, before gently mixing until homogenous. The lysate was then centrifuged again at 15,000 × *g* for 15 min to separate soluble proteins from insoluble proteins. The supernatant was then collected as the bacterial protein extract.

### 2.12 Gel Electrophoresis

EV samples were diluted 1:1 in 2× Laemmli Sample Buffer containing 355 mM 2‐mercaptoethanol. Following dilution, samples were incubated at 95°C for 5 min with shaking. Samples were then loaded onto gels, with 25 µL loaded per well, at equal protein concentrations from BCA quantification. The protein ladder was diluted to a 25% concentration in 2× Laemmli sample buffer without 2‐mercaptoethanol, and 25 µL of this working solution was loaded into the lanes on either side of each gel. Gels were run at 150 V for 30–35 min. Gels were fixed before silver staining was conducted.

### 2.13 Western Blotting

After migration, bacterial and EV proteins were transferred onto 0.45 µm PVDF membranes using the BioRad Mini Trans‐Blot Cell at 150 V for 25 min, with ambient temperatures of 4°C. The membranes were air‐dried and stored between ultra‐thick filter paper, in 4°C, and sealed with dessicants to absorb any excess moisture. Membranes were stained with SYPRO Ruby for total protein quantification. Prior to antibody staining, the membranes were blocked in EveryBlot blocking buffer for 10 min on a shaker at room temperature. All primary and secondary antibody dilutions were also done using this buffer. RecA staining was conducted using anti‐Rad51 antibody as RecA and Rad51 are structurally and functionally similar (Seitz et al. 1998). After the primary incubations, the membranes were washed three times for 15 min with TBS supplemented with 0.1% Tween‐20. The membranes were then incubated with secondary antibody solutions for 1 h. Each secondary antibody solution was also prepared with StrepTactin‐HRP conjugate (1:10,000 dilution) for the visualization of the unstained protein ladder. After the secondary incubation period, the membranes were washed 4× with TBST for 20 min, before being transferred to 5 mL of SuperSignal West Pico PLUS Chemiluminescent Substrate for 5 min. Lastly, all blots were imaged using a ChemiDoc Imager in chemiluminescence mode.

### 2.14 Protein Normalization

All band intensities were calculated in Image Lab version 6.1 software. Each lane was manually fit and each band was detected at the expected molecular weight. Band intensities were normalized to total protein loaded by dividing the target protein band chemiluminescent intensity by the SYPRO Ruby image adjusted lane intensity.

### 2.15 Transmission Electron Microscopy

Three hundred mesh, carbon film copper grids were glow discharged immediately prior to sample loading. Each grid was then loaded with 10 µL of EV sample and covered for 30 min. After the incubation period, the excess liquid was blotted off from each grid using filter paper. Each grid was then spot rinsed six times with deionized water. The excess liquid from each grid was blotted off again before the grids were spot rinsed in a droplet of Nano‐W negative stain. Grids were then suspended on a drop of Nano‐W for 1 min before final blotting. Grids were air dried and then stored away until imaging. All images were captured on a JEOL JEM‐1200EX Electron microscope at a 40,000× magnification.

### 2.16 Statistical Analysis

Three replicates were used for each experiment. Where applicable (BCA Assay, Griess assay), the values of technical duplicates were averaged to obtain the value for each replicate. The values of the three replicates were then used in further analyses. Statistical analyses were carried out in GraphPad Prism. Unless otherwise noted, all values represent mean ± standard deviation. To compare particle size, concentration, protein content, and macrophage activation before and after storage, two‐way ANOVAs were used with matched values for each replicate and adjustment for multiple comparisons to the initial values.

## 3 Results

### 3.1 EV Purity

**FIGURE 1:**
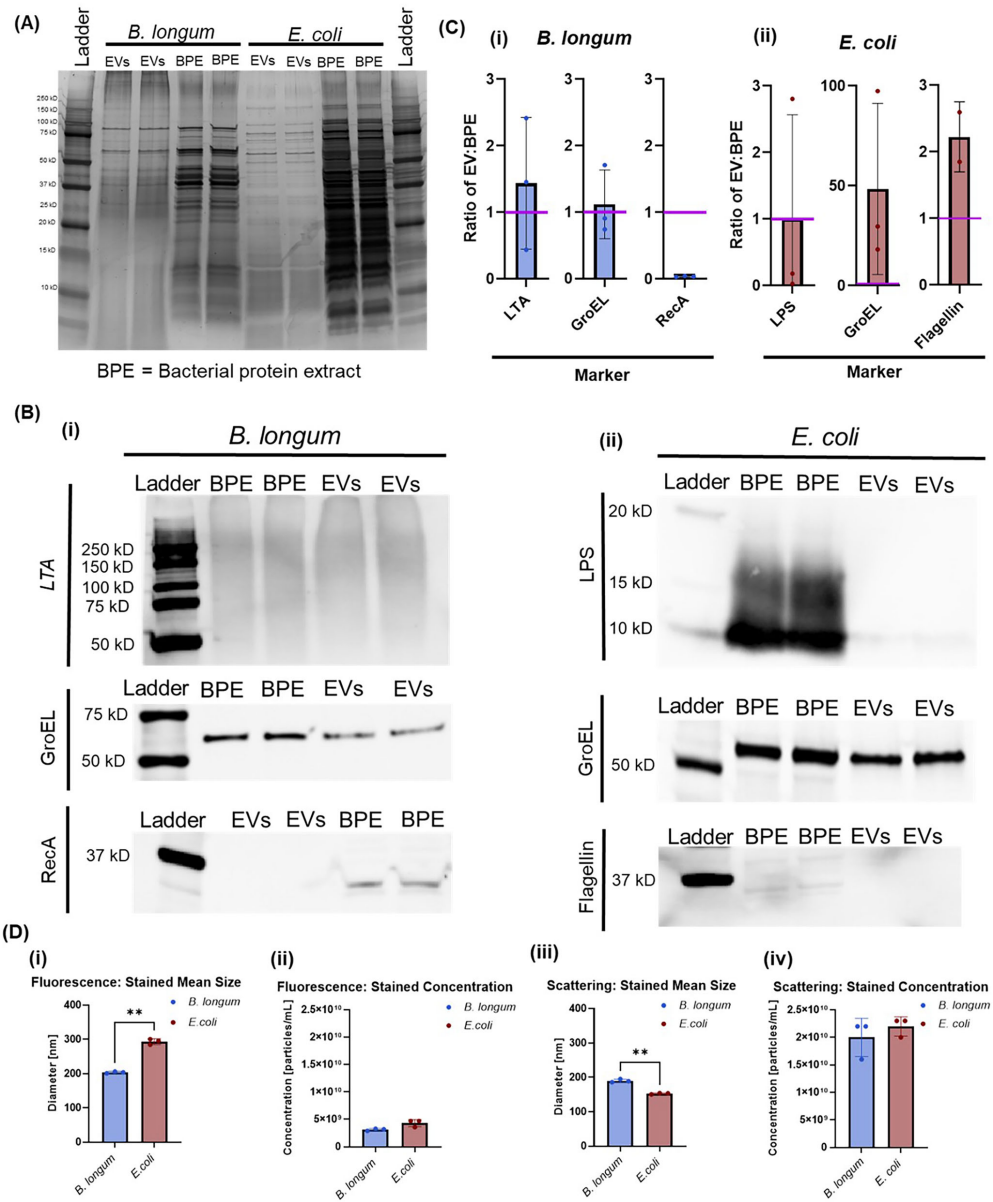
Bacterial EV purity. **(A)** SDS‐PAGE gel image of EVs and bacterial protein extracts (BPE) from the same strains of bacteria. Image is representative from *n* = 3 replicates. The image was cropped and flipped horizontally for ease of interpretation. **(B)** Western blot staining for (i) LTA, GroEL, and RecA on *Bifidobacterium longum* EV and BPE samples and (ii) LPS, GroEL, and Flagellin on *Escherichia coli* EV and BPE samples. Images are representative cropped images from *n* = 3 replicates. The *E. coli* EV GroEL image was flipped horizontally for ease of interpretation. **(C)** Quantification of the ratio of EV to BPE western blot band intensity in each blot for *B. longum* (i) and *E. coli* (ii). *n* = 3 replicates were used, and band intensities were adjusted to total protein loaded in each lane prior to calculation of the ratio. Purple lines represent a ratio of 1, which would indicate that the normalized EV band intensity matched the normalized BPE band intensity. One replicate was excluded for quantification of the *E. coli* Flagellin blots due to high background signal. **(D)** Nanoparticle tracking analysis results from fluorescently‐stained *B. longum* and *E. coli* EVs. Size (i) and concentration (ii) of EVs, as measured on the 488 nm fluorescence channel. Size (iii) and concentration (iv) of EVs, as measured on the 488 nm scattering channel. Data represents *n* = 3 measurements from one sample each for *B. longum* EVs and *E. coli* EVs. Statistical significance was determined using unpaired t tests with Welch's correction.

To evaluate EV purity, EVs were compared to the bacterial strains from which they were isolated. First, SDS‐PAGE was conducted on bacterial protein extracts and EVs from the same strain (Figure 1A, Figure S1). *B. longum* bacterial protein extracts exhibited several bands in the range of 10–250 kDa, while *B. longum* EVs exhibited fewer bands and a reduced intensity in bands that remained. *E. coli* bacterial protein extracts exhibited several bands in the range of 10–250 kDa, and *E. coli* EVs exhibited fewer bands, with reduced intensity in the bands that remained.

Western blotting was conducted for several markers of interest for EVs from each strain. For *B. longum*, the tested markers were lipoteichoic acid (LTA), RecA, and GroEL. LTA, RecA, and GroEL were all identified in the *B. longum* bacterial protein extract samples. LTA and GroEL were identified in the *B. longum* EVs (Figure 1B, Figures S2–S7). Normalized to total protein loaded, *B. longum* EVs had comparable LTA and GroEL to *B. longum* protein extracts and negligible RecA (Figure 1C).

For *E. coli*, the tested western blot markers were lipopolysaccharide (LPS), flagellin, and GroEL. LPS, Flagellin, and GroEL were identified in both *E. coli* bacterial protein extract samples and *E. coli* EVs (Figure 1B, Figures S5–S10). Normalized to total protein loaded, *E. coli* EVs had comparable LPS, higher GroEL, and some increase in Flagellin compared to *E. coli* protein extracts (Figure 1C).

To confirm the membranous nature of the EVs, both *B. longum* and *E. coli* EVs were stained with Alpha Nano Tech EVs Membrane Dye. Using fluorescent nanoparticle tracking analysis (FNTA), the binding of the membrane stain with the EVs was confirmed (Figure 1D). When measured on the 488 nm fluorescence channel, fluorescently stained *B. longum* EVs were found to have a mean particle size of 203.6 ± 2.3 nm and concentration of 3E+09 ± 2E+08 particles/mL, while fluorescently stained *E. coli* EVs were found to have a mean particle size of 293.5 ± 6.7 nm and concentration of 4E+09 ± 5E+08 particles/mL. When measured on the 488 nm scattering channel, fluorescently stained *B. longum* EVs were found to have a mean particle size of 189.9 ± 3.8 nm and concentration of 2E+10 ± 2.8E+09, while fluorescently stained *E. coli* EVs were found to have a mean particle size of 152.7 ± 1.5 nm and concentration of 2.2E+10 ± 1.4E+09.

### 3.2 EV Size and Concentration

**FIGURE 2:**
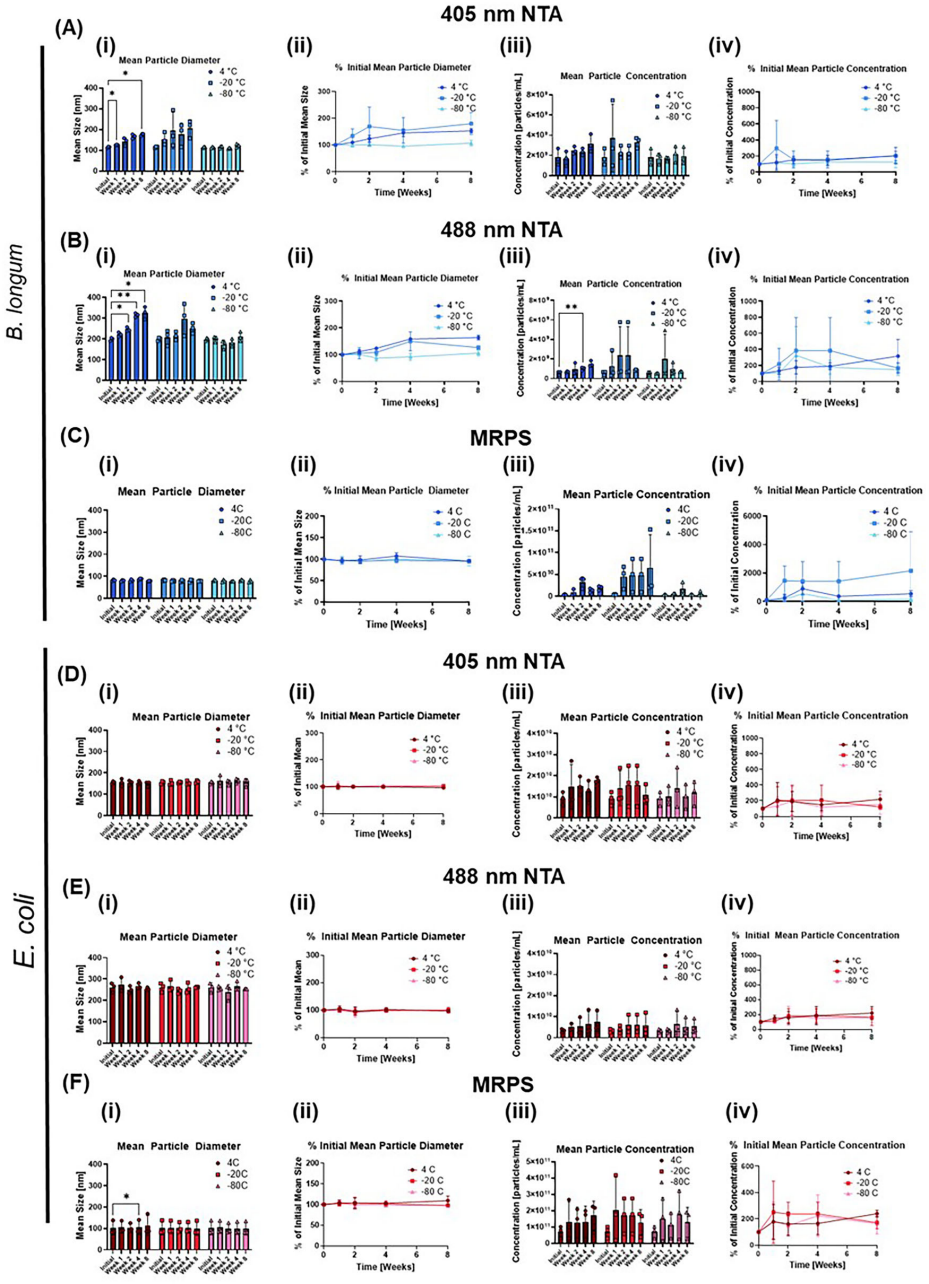
Bacterial EV particle size and concentration before and after storage. For each graph, data for the initial condition is repeated across the three temperature conditions because each replicate was separated into several aliquots, which were then stored at the different temperatures. *n* = 3 replicates were used for each experiment. **(A)** *Bifidobacterium longum* EV size (i‐ii) and concentration (iii‐iv) when measured with NTA at a wavelength of 405 nm. **(B)** *B. longum* EV size (i‐ii) and concentration (iii‐iv) when measured with NTA at a wavelength of 488 nm. **(C)** *B. longum* EV size (i‐ii) and concentration (iii‐iv) when measured with MRPS. **(D)** *Escherichia coli* EV size (i‐ii) and concentration (iii‐iv) when measured with NTA at a wavelength of 405 nm. **(E)** *E. coli* EV size (i‐ii) and concentration (iii‐iv) when measured with NTA at a wavelength of 488 nm. **(F)** *E. coli* EV size (i‐ii) and concentration (iii‐iv) when measured with MRPS. Statistical significance was determined by two‐way ANOVA assays with matched values for each replicate and adjustment for multiple comparisons to the initial values. Asterisks in panels i and iii represent statistical significance with respect to the initial values.

The bacteria from which EVs were isolated had a final wellplate OD600 of 0.48 ± 0.12 AU for *B. longum* and 0.47 ± 0.11 AU for *E. coli*, which corresponded to traditional cuvette‐based OD600 measurements of 1.23 ± 0.10 AU and 1.11 ± 0.10 AU, respectively (Figure S11). Immediately after EV isolation, bacterial EV size and concentration were evaluated using both nanoparticle tracking analysis (NTA) and microfluidic resistive pulse sensing (MRPS). NTA, which uses a scattering‐based technique, can detect different ranges of particle sizes based on laser wavelength used, with higher wavelengths biased toward larger particle sizes. On the other hand, MRPS detects pulses in resistance as particles pass through a constriction. Using 405‐nm laser excitation, NTA revealed mean sizes of 115.2 ± 3.7 nm and 154.6 ± 6.7 nm for freshly isolated *B. longum* and *E. coli* EVs, respectively (Figure 2A,D). Using 488‐nm laser excitation, NTA revealed mean sizes of 198.8 ± 8.0 nm and 260.7 ± 21.8 nm for freshly isolated *B. longum* and *E. coli* EVs, respectively (Figure 2B,E). Meanwhile, MRPS measurements of freshly isolated *B. longum* and *E. coli* EVs revealed mean sizes of 81.8 ± 3.3 nm and 101.8 ± 31.5 nm, respectively (Figure 2C,F).

After just 4 weeks of storage at 4°C, a significant increase in NTA mean particle size is observed for *B. longum* EVs measured with the 488 nm laser.This trend continues with 8 weeks of storage at the 4°C temperature, regardless of the measurement wavelength used. In contrast, storage at other temperatures did not lead to significant changes in *B. longum* EV size over the 8‐week period, although the −80°C storage temperature appeared to lead to the most stable mean particle size. For *E. coli* EVs, no significant changes in NTA size were observed over the 8‐week period for any storage temperature. MRPS size measurements showed no significant changes for either *B. longum* or *E. coli* EVs regardless of storage temperature, with the exception of *E. coli* EVs stored at 4°C for 4 weeks.

When particle concentration was quantified using NTA with a 488 nm laser, we observed significant differences between particle concentrations of initial *B. longum* EVs and those stored at 4°C for 4 weeks. However, this trend does not hold true at the 8‐week timepoint, or with the use of the 405 nm laser. There were no significant changes in *E. coli* EV concentration for either NTA laser wavelength. With MRPS quantification of particle concentration, there were no significant changes in concentration before and after EV storage for either the *B. longum* or *E. coli* EVs.

### 3.3 EV Functionality

**FIGURE 3:**
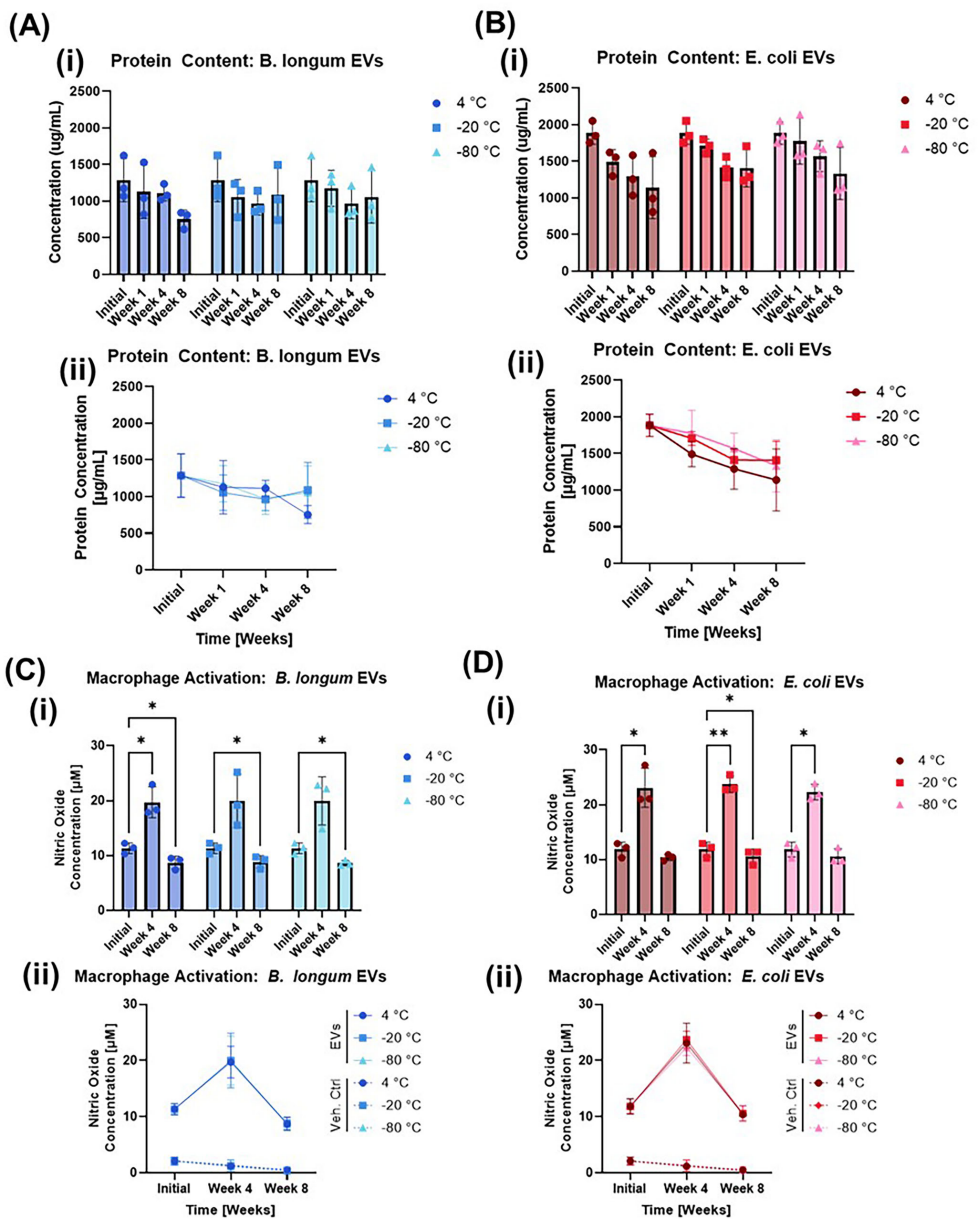
Protein concentration and macrophage activation by EVs. For each graph, data for the initial condition is repeated across the three temperature conditions because each replicate was separated into several aliquots, which were then stored at the different temperatures. *n* = 3 replicates were used for each experiment. **(A)** Bar graphs (i) and line graphs (ii) of *Bifidobacterium longum* EV protein concentration before and after storage, as quantified by BCA assays. Statistical significance was determined by two‐way ANOVA assays with matched values for each replicate and adjustment for multiple comparisons to the initial values. There are no significant changes in protein concentration observed with time. **(B)** Bar graphs (i) and line graphs (ii) of *Escherichia coli* EV protein concentration before and after storage, as quantified by BCA assays. Statistical significance was determined by two‐way ANOVA assays with matched values for each replicate and adjustment for multiple comparisons to the initial values. There are no significant changes in protein concentration observed with time. **(C)** Bar graphs (i) and line graphs (ii) of RAW264.7 macrophage activation by *B. longum* EVs. A dose of 12.5% (v/v) EV suspension was used. Statistical significance was determined by two‐way ANOVA assays with matched values for each replicate and adjustment for multiple comparisons to the initial values. Storage at 4°C for 4 or 8 weeks significantly altered macrophage activation, as did storage at −20°C or −80°C for 8 weeks. **(D)** Bar graphs (i) and line graphs (ii) of RAW264.7 macrophage activation by *E. coli* EVs. A dose of 12.5% (v/v) EV suspension was used. Statistical significance was determined by two‐way ANOVA assays with matched values for each replicate and adjustment for multiple comparisons to the initial values. Storage at 4°C, −20°C, or −80°C for 4 weeks significantly increased macrophage activation.

To evaluate the ability of the different storage conditions to retain EV functionality, BCA and Griess assays were conducted. First, BCA assays were used to quantify EV protein content. Before storage, *B. longum* EVs had an average protein content of 1286.4 ± 295.4 µg/mL (Figure 3A), and *E. coli* EVs had an average protein content of 1883.9 ± 151.9 µg/mL (Figure 3B). No statistically significant changes in protein concentration were observed for either *B. longum* or *E. coli* EVs over the 8‐week storage period. These results confirm that bacterial EV protein concentration is stable after sample storage.

Next, RAW264.7 macrophages were treated with different concentrations of EVs before and after storage. LPS in gram‐negative EVs and LTA in gram‐positive EVs are known to lead to macrophage activation. Prior to EV storage, we observed a dose‐dependent macrophage activation in response to 24 h of incubation with EVs (Figure S12), with a nitric oxide concentration of 11.3 ± 1.0 µM for macrophages treated with culture medium containing 12.5% v/v *B. longum* EVs (Figure 3C), and a nitric oxide concentration of 11.8 ± 1.4 µM for macrophages treated with culture medium containing 12.5% v/v *E. coli* EVs (Figure 3D). The vehicle control led to minimal macrophage activation compared to the EV suspensions, with a nitric oxide level of only 2.1 ± 0.7 µM for the highest concentration of vehicle control (Figure S13).

Interestingly, a marked increase in macrophage activation capability was observed for both *B. longum* and *E. coli* EVs after EV storage for 4 weeks, regardless of storage temperature. This increase was statistically significant for *B. longum* EVs stored at 4°C, and for *E. coli* EVs stored at −20°C or −80°C. After 8 weeks of storage, macrophage activation levels fell, with a significantly lower concentration of nitric oxide for *B. longum* EVs stored at all temperatures. For *E. coli* EVs stored for 8 weeks, macrophage activation decreased from the initial measurements only for samples stored at −20°C, but not for those stored under the other conditions.

**FIGURE 4:**
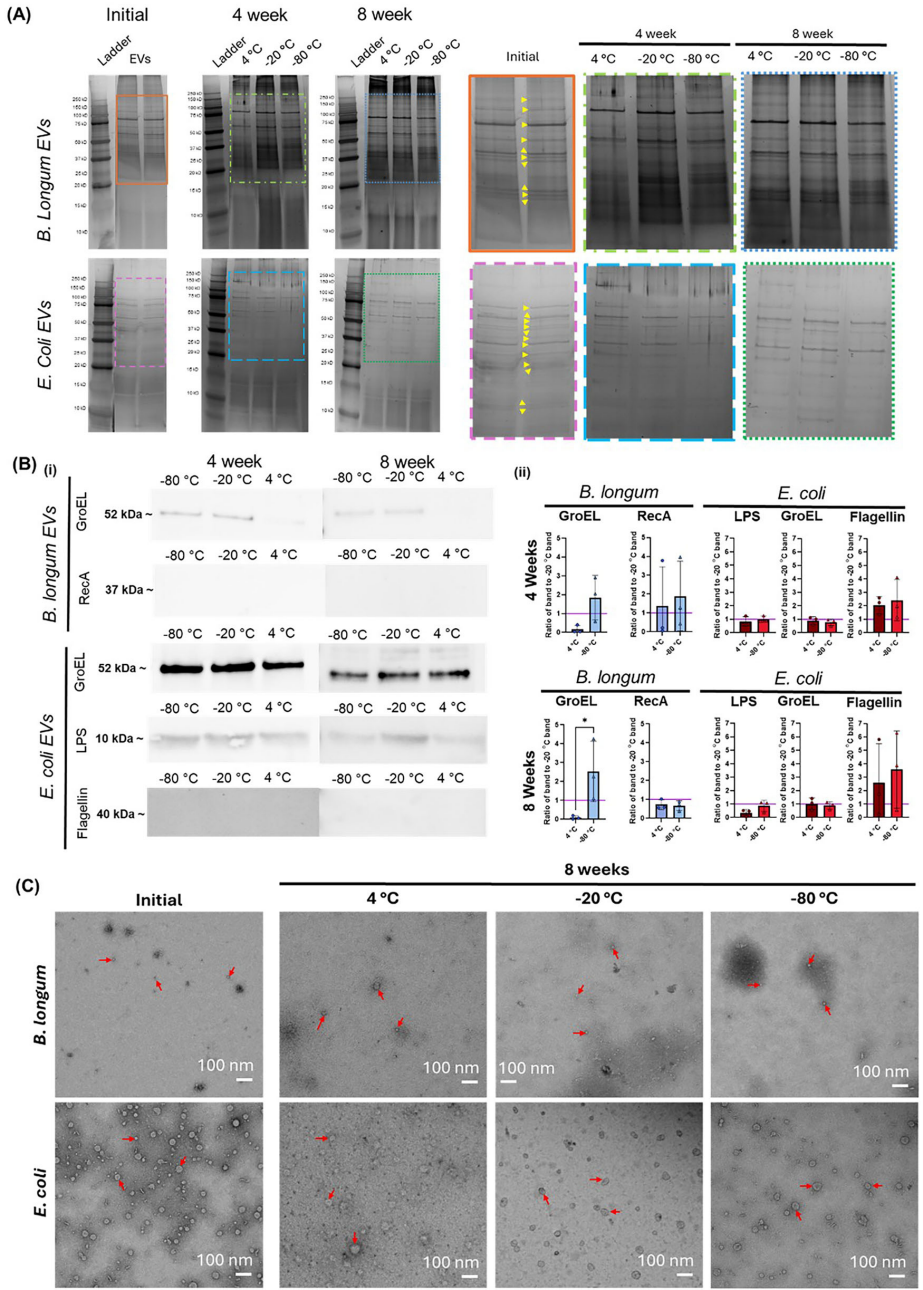
**(A)** SDS PAGE results for samples before and after storage. Images are cropped representative images selected from *n* = 3 replicates. **(B) (i)** Western blot bands after 4 and 8 weeks of storage at 4°C, −20°C, and −80°C. The continued presence or absence of bands is observed. Images are cropped representative images selected from *n* = 3 replicates. Week 4 *Bifidobacterium longum* GroEL, Week 4 *Escherichia coli* LPS, Week 4 *E. coli* Groel, and Week 8 *Bifidobacterium longum* RecA blots were flipped horizontally for ease of interpretation. **(ii)** Ratio of western blot band intensities for EVs stored at 4°C, and −80°C, versus −20°C. Ratios were calculated after the intensity of each band was normalized to the total protein loaded in that lane. Purple lines reflect a ratio of 1, which would indicate that the samples stored at 4°C or −80°C maintained the same normalized band intensity as samples stored at −20°C. Statistical significance was determined using paired and ratio paired t tests. **(C)** Transmission Electron Microscopy images of samples before and after storage. Images are representative images selected from three replicate experiments. Red arrows point to examples of EVs.

To further evaluate the protein content of EVs post‐storage, we conducted SDS gel electrophoresis on EV samples before and after storage (Figure 4A). The direct application of EV samples to gels without the need for prior protein extraction has previously been demonstrated by others (Santucci et al. 2019). Before storage, *B. longum* EVs exhibited several protein bands in the range of 20–250 kDa, with the most intense of the bands lying between 75 and 100 kDa. Four bands were present between 50 and 75 kDa, with three of them being very close to each other in molecular weight. Three other bands that were close to each other in molecular weight were observed between 37 and 50 kDa. *E. coli* EVs also exhibited several protein bands in the range of 20–250 kDa, with the most intense bands close to 100, 75, and 50 kDa. Several fainter bands were also visible at a variety of molecular weights between 25 and 150 kDa. After storage, a qualitative reduction in intensity of some faint bands, along with increased protein smearing between bands is observed. Markers used to determine EV purity were again probed with western blotting after 4 and 8 weeks of storage (Figure 4B). LTA was excluded from these analyses because we observed differences in whether a band or a smear was detected across antibody lots. Qualitatively, at both the 4 week and 8 week timepoint *B. longum* EV GroEL bands appeared fainter for EVs stored at 4°C versus EVs stored at lower temperatures. LPS bands also appeared fainter for *E. coli* EVs stored at 4°C for 8 weeks versus EVs stored at lower temperatures. Within each blot, a ratio of band intensity (normalized to total protein loaded) for the 4°C and −80°C conditions to the band intensity (normalized to total protein loaded) of −20°C condition were calculated. At the 4 week timepoint, none of these ratios were significantly different for a storage temperature of −80°C compared to a storage temperature of 4°C. At the 8 week timepoint, only the *B. longum* GroEL ratio was significantly different for a storage temperature of −80°C compared to a storage temperature of 4°C.

Bacterial EV structure was also evaluated using transmission electron microscopy (Figure 4C). Prior to storage, *B. longum* EVs were found to be smaller in size and relatively sparse in appearance compared to *E. coli* EVs. Despite this, bacterial EVs from both *B. longum* and *E. coli* were visualized with transmission electron microscopy after 8 weeks of storage at 4°C, −20°C, or −80°C. EV appearance after storage remained similar to appearance prior to storage. Qualitatively, we observed that locating the samples on the TEM grids proved more challenging in the post‐storage conditions, with increased debris observed.

## 4 Discussion

Due to the relative newness of studies focused on bacterial EVs compared to those focused on mammalian EVs, it is important to establish guidelines for their storage to ensure their biological functions are effectively preserved. In this work we have sought to identify appropriate bacterial EV storage conditions as has been extensively studied for mammalian EVs. By tracking changes in EV size, concentration, protein content, and macrophage activation over time after storage at −80°C, −20°C, and 4°C, we were able to determine the impact of storage at these three temperatures on EV integrity.

EV identity was first confirmed using several techniques. SDS PAGE revealed clear differences in bands between bacterial EVs and bacterial protein extracts. In western blotting experiments, the presence of LTA and GroEL, as well as the absence of RecA, were used to confirm *B. longum* EV purity. Gram‐positive EVs are known to contain LTA (Bose et al. 2020), which is also present in gram‐positive bacteria. We selected RecA as a negative marker for *B. longum* EVs based on our prior proteomic studies (unpublished data), and because RecA is a cytoplasmic rather than membrane protein (Cox 2007). GroEL was selected because it has been used as a marker of EV purity, and is also present in gram‐positive Bifidobacterium (Ventura et al. 2004). LPS is known to be present in gram‐negative bacteria and their EVs (Ñahui Palomino et al. 2021; Fujihara et al. 2003). Flagellin is considered a bacterial cell contaminant in *E. coli* EV preparations, with lower levels of flagellin considered as an indicator of greater EV purity (Hong et al. 2019). GroEL has been commonly used as a marker for *E. coli* EV purity, although recent reports indicate that GroEL enrichment may be associated with crude EV samples (Hong et al. 2019). The presence of LPS and an enrichment of GroEL in the *E. coli* EV samples were used to confirm *E. coli* EV purity. Nevertheless, quantitative analyses revealed an enrichment of Flagellin in the *E. coli* EV samples. Such enrichment may at least partially be due to the departure from the logarithmic growth phase for *E. coli* after 24 h in culture (Figure S14), representing a limitation in our studies.

In fluorescent NTA experiments, binding of a membrane dye with both *E. coli* and *B. longum* EVs was observed, confirming the membranous nature of the EV samples as a further measure of their identity. *E. coli* EVs exhibited a greater mean size than *B. longum* EVs. This matches with our observations from scattering‐based measurements of unstained EVs. However, it is in direct contrast to the results from scattering‐based measurements of the fluorescently‐stained EVs, which found that *E. coli* EVs had a smaller particle size than *B. longum* EVs. This indicates a potential effect of the fluorescent EV stain on particle scattering and therefore scattering‐based detection of particle size.

Looking at EV size and concentration alone, few changes were observed as a result of storage. Only storage at 4°C appeared to lead to significant changes in EV size after 4–8 weeks. An increase in particle size could potentially be attributed to particle agglomeration or previously‐reported fusion phenomena exhibited by EVs (Gelibter et al. 2022). Additionally, bacterial EV size was found to be highly dependent on measurement technique, which further emphasizes the importance of disclosing full experimental details to put EV size measurements in context when comparing across different studies or bacterial strains. The lower particle sizes measured by MRPS compared to NTA are a result of the differences in these measurement techniques, where NTA is a scattering‐based technique and MRPS is a resistance‐based technique. NTA uses light scattered by the nanoparticles to track the Brownian motion of the particles, ultimately using this information to calculate the diffusion coefficient and then the particle size (Comfort et al. 2021). Lower‐wavelength NTA measurements are typically known to result in the measurement of smaller particles, while higher‐wavelength NTA measurements are typically known to result in the measurement of larger particles. Thus, the selection of NTA laser wavelength plays an important role in the results. On the other hand, a failure of NTA instruments to report peak EV diameters less than 60 nm has previously been reported (Bachurski et al. 2019). In MRPS, a fluidic resistor and nanoconstriction form a voltage divider. As particles pass through the nanoconstriction, the change in electrical resistance is measured via the voltage divider as a voltage change (Cimorelli et al. 2021). This is proportional to the particle volume and is used to calculate particle size. One limitation of MRPS is that a surfactant such as Tween 20 must be used to enable the sample to flow through the cartridge microchannels, and these surfactants may have an effect on EV integrity (Cimorelli et al. 2021). At the same time, MRPS size measurements are limited by the size of the constrictions through which the EVs must pass. In this case, we used MRPS cartridges that could measure particles in the range of 65–400 nm. Thus, while the MRPS particle sizes were smaller than the NTA‐detected particle sizes, particles below 65 nm would not be detected or incorporated into the mean size calculations. Despite the limitations in minimum particle size measurable by both of these techniques, the trend of a larger particle size for *E. coli* EVs compared to *B. longum* EVs remained true across all the unstained EVs, regardless of the measurement technique or wavelength used. The greater particle concentrations observed by MRPS can also be attributed to a vast population of small vesicles that were beyond the scope of the NTA's camera sensitivity. The idea of EV subpopulations, differentiated by size, is further supported by the noticeable shifts between the different laser wavelengths in NTA. Additionally, the greater spike in concentration measurements across the two methods could potentially indicate a lower refractive index of *B. longum* EVs in comparison to *E. coli* EVs. Indeed, the refractive index of particles in suspension is known to impact light scattering, thus affecting NTA results (Filipe et al. 2010; Tian et al. 2016). Altogether, this highlights the need for nanoparticle characterization to be multidimensional, as each technique provides different insights in bacterial EV studies.

However, functional evaluation through macrophage activation studies and gel electrophoresis suggests changes in EV biological function that are not obvious from size and concentration studies. An overall increase (at 4 weeks of storage) and subsequent decrease (at 8 weeks of storage) in macrophage activation for EVs from both *B. longum* and *E. coli* suggests EV immune activation is affected by storage, regardless of storage temperature. This suggests changes in immunomodulatory properties of bacterial EVs with storage. Gram‐positive EVs contain lipoteichoic acid (Bose et al. 2020), whereas gram‐negative bacterial EVs contain lipopolysaccharide (Ñahui Palomino et al. 2021; Fujihara et al. 2003). Both LPS and LTA are immunostimulatory factors that can trigger macrophage activation (Fujihara et al. 2003; Grunfeld et al. 1999). The unexpected increase of macrophage activation after 4 weeks of storage might perhaps be due to greater accessibility of LPS, LTA, or other stimulatory molecules if EV structural integrity is compromised in the early weeks of storage. At the lower storage temperatures, such structural disruptions could potentially be the result of the single freeze‐thaw cycle to which the EVs were exposed. Previous studies have studied the effects of freeze‐thawing on EVs, and have found varying impacts on particle size, particle yield, and structure. For example, some studies have found that even a single freeze‐thaw cycle could greatly decrease particle yield (Gelibter et al. 2022), and additional cycles could lead to increasing particle size (Gelibter et al. 2022; Cheng et al. 2019; Wu et al. 2021). Such changes may be attributed to particle aggregation and swelling due to freezing, which has been supported by multiple studies (Gelibter et al. 2022; Lőrincz et al. 2014; Maroto et al. 2017). Ice crystals generated by freezing can also cause physical damage to the lipid membrane of EVs in the form of tearing (Qin et al. 2020). It should, however, be noted that certain types of EVs have shown exceptional stability under freeze‐thaw conditions (Qin et al. 2020; Trenkenschuh et al. 2022).

Meanwhile, a reduction in EV macrophage activation after 8 weeks of storage suggests potential degradation of the proteins that were released during the early weeks of EV storage. These observations suggest that bacterial EV immunomodulatory properties can change during storage, and this can lead to nonlinear changes in macrophage activation with storage time. Previous work has demonstrated that rough LPS, which lacks the extended O‐chain, is internalized more efficiently by macrophages, than smooth LPS, highlighting how structural differences in LPS organization influence cell uptake and immune activation (Sali et al. 2019). Consequently, the aggregation of EVs during freeze‐thaw cycles may alter the accessibility of immunoactive LPS, potentially leading to phase like shifts in macrophage stimulation that manifest as nonlinear activation profiles over time. Prior studies have demonstrated a reduction in antibacterial properties of EVs derived from human neutrophilic granulocytes after 28 days of storage (Lőrincz et al. 2014). In that context, other changes in EV function, such as the change in macrophage activation that we observed for our bacterial EVs, are not entirely unexpected. It is important for such potential changes to be considered and characterized for EVs from various strains of bacteria, as it relates to the specific applications for which those EVs will be used.

Though both *E. coli* EVs and *B. longum* EVs displayed similar trends, differences were observed between the EVs isolated from the two species regarding particle size stability. These differences may potentially be attributed to the differences in biogenesis and structure between EVs produced by gram‐positive bacteria and those produced by gram‐negative bacteria. The mechanism of EV biogenesis in gram‐positive bacteria is not fully understood (Briaud and Carroll 2020). EV formation and release in gram‐negative bacteria relies on vesicle budding and subsequent pinching off from the membrane (Jan 2017; Brown et al. 2015; Roier et al. 2016). The presence of LPS in the gram‐negative bacterial outer membrane lends greater stability to the bacterial outer membrane compared to the cytoplasmic membrane (Coleman and Smith 2014). Several genes involved in vesiculogenesis for gram‐negative bacteria have been identified by others, and various mechanisms for the formation of EVs in gram negative bacteria have been proposed (Jan 2017). In one such mechanism, vesicle formation is triggered due to a decrease in cross‐linking between the thin peptidoglycan layer in gram‐negative cells and the outer membrane (Jan 2017). Another proposed mechanism suggests that accumulation of phospholipids in the outer leaflet of the bacterial outer membrane results in outward bulging of the membrane and subsequent vesicle formation (Roier et al. 2016). Stress appears to stimulate the production of EVs (Jan 2017). Under conditions of severe stress in which DNA damage occurs, the peptidoglycan layer in gram‐negative bacteria may thin, leading the cell to burst and release EVs in a process called explosive cell lysis (Briaud and Carroll 2020). Similar mechanisms are possible in gram‐positive bacteria, but EV release is more complicated due to the impediment of the gram‐positive cell wall (Briaud and Carroll 2020). It has been proposed that the mechanism may share similarities with EV release in mycobacteria and fungi, which also have thick cell walls (Brown et al. 2015). Researchers have suggested that gram‐positive bacterial EV formation and release are regulated by a complex array of genes, although a specific pathway has not been identified (Briaud and Carroll 2020; Brown et al. 2015). One idea contends that turgor pressure may force the EVs through pores in the cell wall (Briaud and Carroll 2020; Brown et al. 2015). Another hypothesis suggests that reduced crosslinking of the peptidoglycan wall can allow escape of EVs from the cell (Briaud and Carroll 2020). These distinct mechanisms of formation may have an impact on the structural composition and stability of the EVs they produce. In addition to the differences between gram‐positive and gram‐negative bacteria, the conditions of growth can also have effects on the EVs produced. Studies have shown that bacterial EVs isolated from planktonic growth conditions, where bacteria are free‐floating, differ from biofilm‐derived EVs in traits such as particle size, surface charge, cargo, and even the roles they fulfill (Johnston et al. 2023; Leiva‐Sabadini et al. 2025). The bacteria in this study were grown under planktonic conditions, and further study should be done in order to characterize the impact of storage on EVs isolated from biofilms versus those isolated from planktonic bacteria.

EV protein concentration, as measured by BCA assays, did not undergo any statistically significant changes. We further evaluated changes in SDS PAGE protein bands with EV storage. The observation of reduced intensity in some faint protein bands, combined with increased protein smearing between bands, suggests some potential degradation of EV‐associated proteins during storage. In western blotting experiments, qualitative reductions in *B. longum* GroEL and *E. coli* LPS band intensities for samples stored at 4°C compared to those stored at −20°C or −80°C were observed, suggesting that specific proteins of interest were degrading with EV storage. Despite this, there were few significant differences when comparing the total‐protein‐loaded‐normalized band intensity ratios (4°C: −20°C compared to −80°C: −20°C). Here, we faced limitations in meaningful quantification of these blots due to the inability to run initial and post‐storage EVs of the same replicate on a single membrane.

The relative sparseness of *B. longum* EVs on the TEM grids compared to *E. coli* EVs on the TEM grids is possibly because the bacterial OMV isolation kit used for isolation of the EVs is optimized for gram‐negative EV isolation. Additionally, the observation of increased debris on TEM grids prepared with post‐storage EVs was possibly a result of EV degradation or disruptions to the EV membranes.

Overall, it is clear that the use of fresh bacterial EVs would be preferable to the use of stored EVs. If stored bacterial EVs are to be used, the use of frozen rather than refrigerated EVs would be preferable. However, changes in bacterial EV functionality such as protein content and macrophage activation can be expected with storage. Importantly, the functional stability of EVs does not closely correlate with size and concentration measurements. Although *E. coli* EVs exhibited greater stability in size and concentration than *B. longum* EVs, both *B. longum* and *E. coli* EVs exhibited changes in macrophage activation with storage. For bacterial EVs, evaluating the effects of storage conditions on specific strains and applications of interest would provide the best understanding of EV stability for each use case. Further work is needed to evaluate the effects of different storage buffers. Although there are some commercially available EV storage buffers for mammalian EVs, it is not clear whether these would mitigate the effects of storage on bacterial EV stability, and their suitability for such usage merits further investigation.

## 5 Conclusion

In conclusion, storage of bacterial EVs can impact EV properties and functionality. The use of different measurement parameters, such as wavelength on NTA, also impact results. These findings confirm the importance of uniform and detailed reporting of experimental procedures regarding bacterial EV storage and measurement, as well as the need to further characterize the impacts of storage on bacterial EVs. This study is limited in that it examines EVs derived from two specific species of bacteria, which cannot wholly encompass the biological variety of all bacterial EVs. Additionally, only one EV isolation method, the use of a kit, was tested. Further research must be conducted to evaluate the post‐storage behaviour of EVs isolated through other methods such as ultracentrifugation, size exclusion chromatography, or tangential flow filtration. Consideration should also be given to the bacterial growth phase at the time of EV isolation, which we have not examined in detail in this work. Furthermore, EVs derived from biofilms were not examined in this study; given the differences between planktonic and biofilm‐derived EVs, this is a gap that must be addressed in future work. Further studies must be done, with a greater variety of bacterial species and culture conditions, to achieve a more complete characterization of the impact of storage on bacterial EVs. The impact of additional storage conditions, such as storage buffer, must also be assessed.

## Author Contributions

**Anagha Rama Varma**: conceptualization, data curation, formal analysis, visualization, writing – original draft, methodology, investigation, writing – review and editing. **Frank Borris**: conceptualization, data curation, formal analysis, visualization, writing – review and editing, methodology, investigation. **Parnika Kant**: investigation. **Kaitlyn Sadtler**: resources, writing – review and editing, conceptualization, methodology. **Parinaz Fathi**: conceptualization, formal analysis, visualization, writing – original draft, supervision, writing – review and editing, project administration, funding acquisition, resources, methodology.

## Conflicts of Interest

 The authors declare no conflicts of interest.

## Supporting information




**Supplementary Materials**: jex270143‐sup‐0001‐SuppMat.docx

## Data Availability

The data that support the findings of this study are available from the corresponding author upon reasonable request.
