# Supplementary material for: Long‐Term Stability of Bacterial Extracellular Vesicles Stored at Different Temperatures
Source: J Extracell Biol. 2026 May 25;5(5):e70143. doi: 10.1002/jex2.70143 (PMC13240203; doi:10.1002/jex2.70143)
Supplement: Supplementary file 1 — Supplementary Materials: jex270143‐sup‐0001‐SuppMat.docx [file JEX2-5-e70143-s001.docx]

Supplementary information for

**Long-term Stability of Bacterial Extracellular Vesicles Stored at Different Temperatures**

*Anagha Rama Varma,‡ ^1, 2, 3^ Frank Borris,‡ ^1, 2, 3^ Parnika Kant, ^1, 2, 3^ Kaitlyn Sadtler,^2, 3^ Parinaz Fathi ^1, 2, 3^**

^1^ NanoEngineering and MicroPhysiological Systems Section (NEMPSS), National Institute of Biomedical Imaging and Bioengineering (NIBIB), National Institutes of Health (NIH), Bethesda MD, USA

^2^ Section on Immunoengineering, National Institute of Biomedical Imaging and Bioengineering (NIBIB), National Institutes of Health (NIH), Bethesda MD, USA

^3^ Biomedical Engineering and Technology Acceleration Center, National Institute of Biomedical Imaging and Bioengineering, Bethesda, MD, USA

*‡* Denotes Equal contribution

*Corresponding author: Parinaz.fathi@nih.gov

**Table of Contents**

Supplementary Figures…………………………………………………………………………………………..3

Supplemental Methods…………………………………………………………………………………………17

**Supplementary Figures**

**
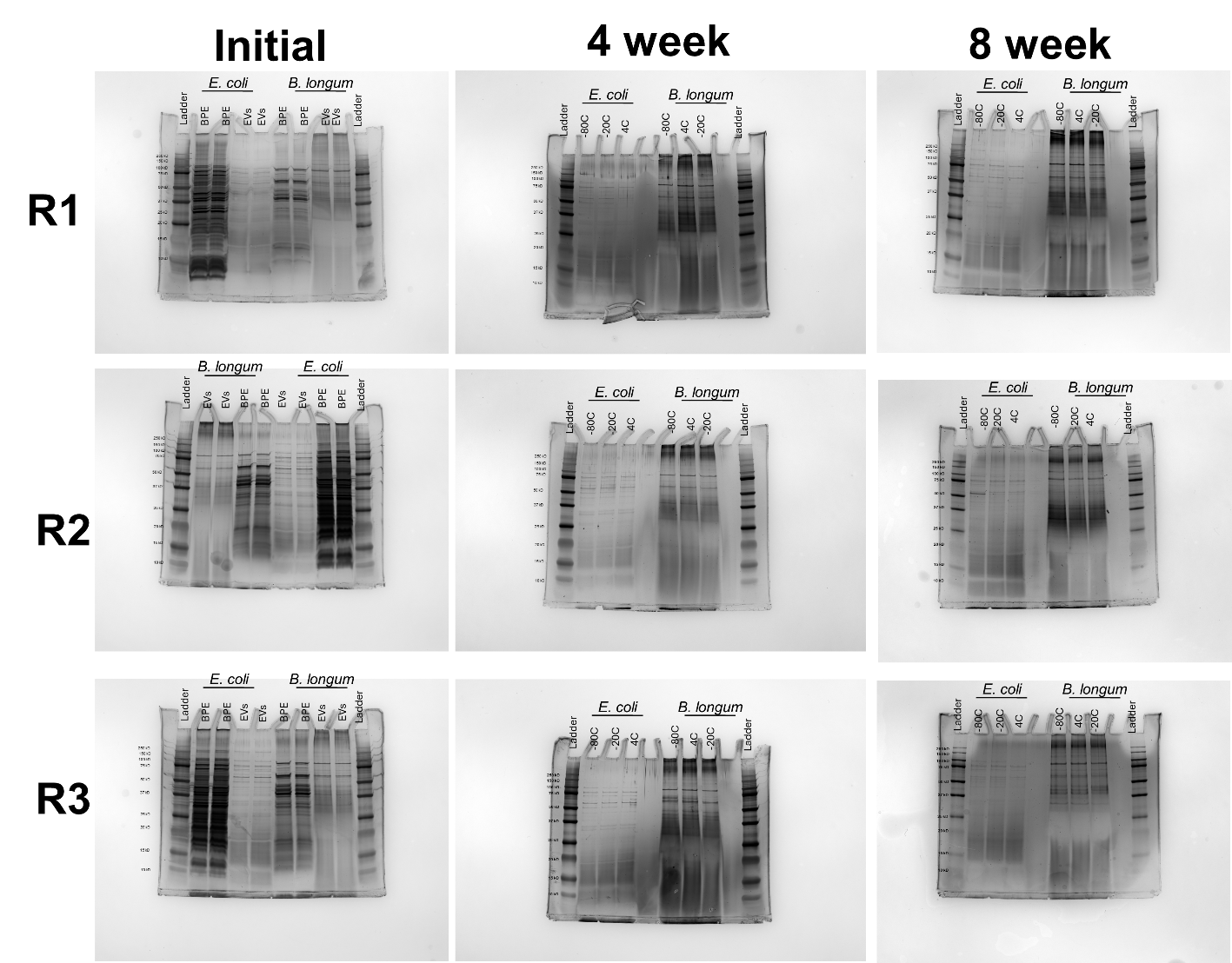
**

**Figure S1.** Silver-stained SDS PAGE gel images at the initial, 4-week, and 8-week timepoints. Initial images include both EVs and BPEs, while 4-week and 8-week timepoints include EVs after storage at 4 °C, -20 °C, and -80 °C.

**
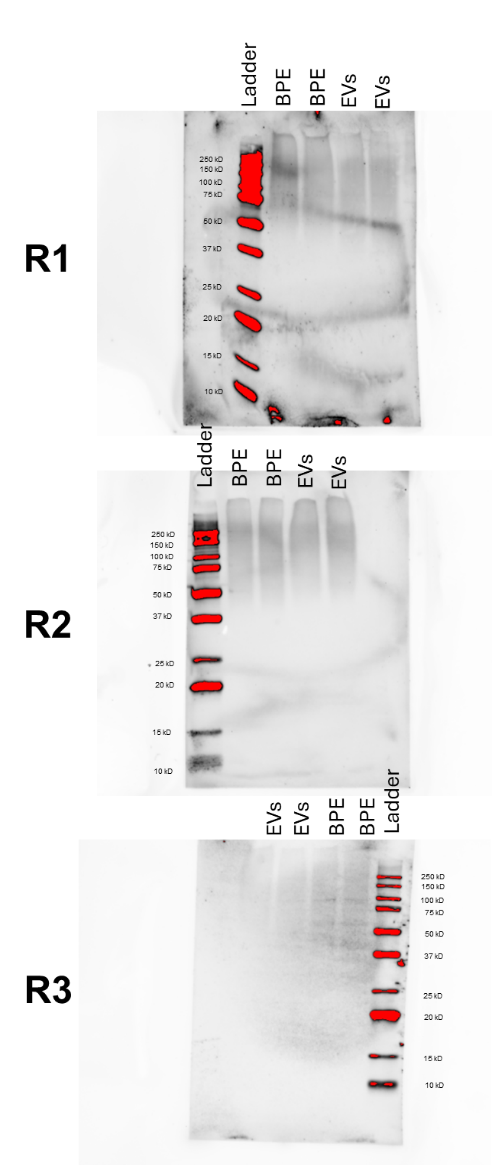
**

**Figure S2.** *B. longum* LTA western blots at initial timepoint. Iimages include both EVs and BPEs.


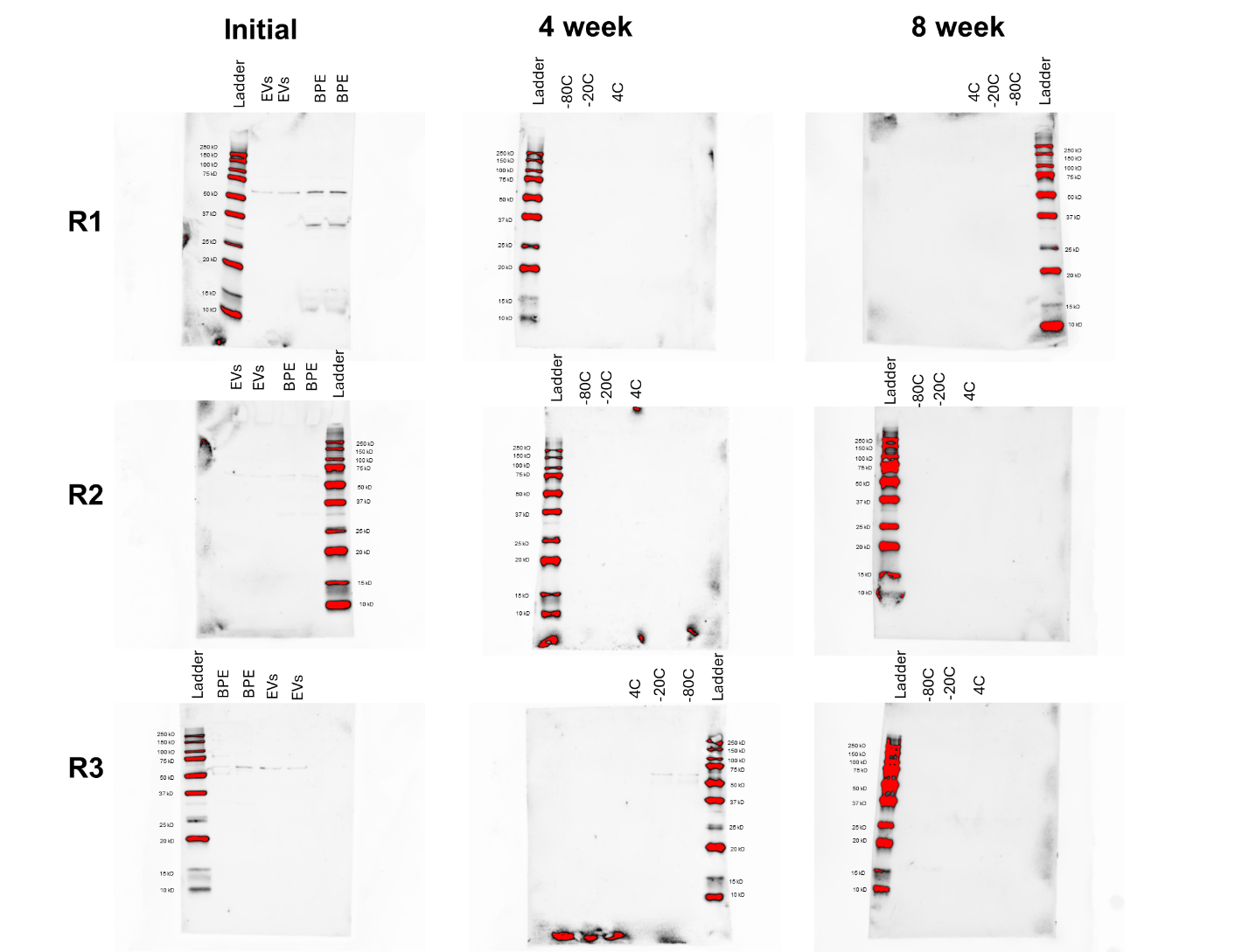


**Figure S3.** *B. longum* RecA western blots at initial, 4-week, and 8-week timepoints. Initial images include both EVs and BPEs, while 4-week and 8-week timepoints include EVs after storage at 4 °C, -20 °C, and -80 °C.


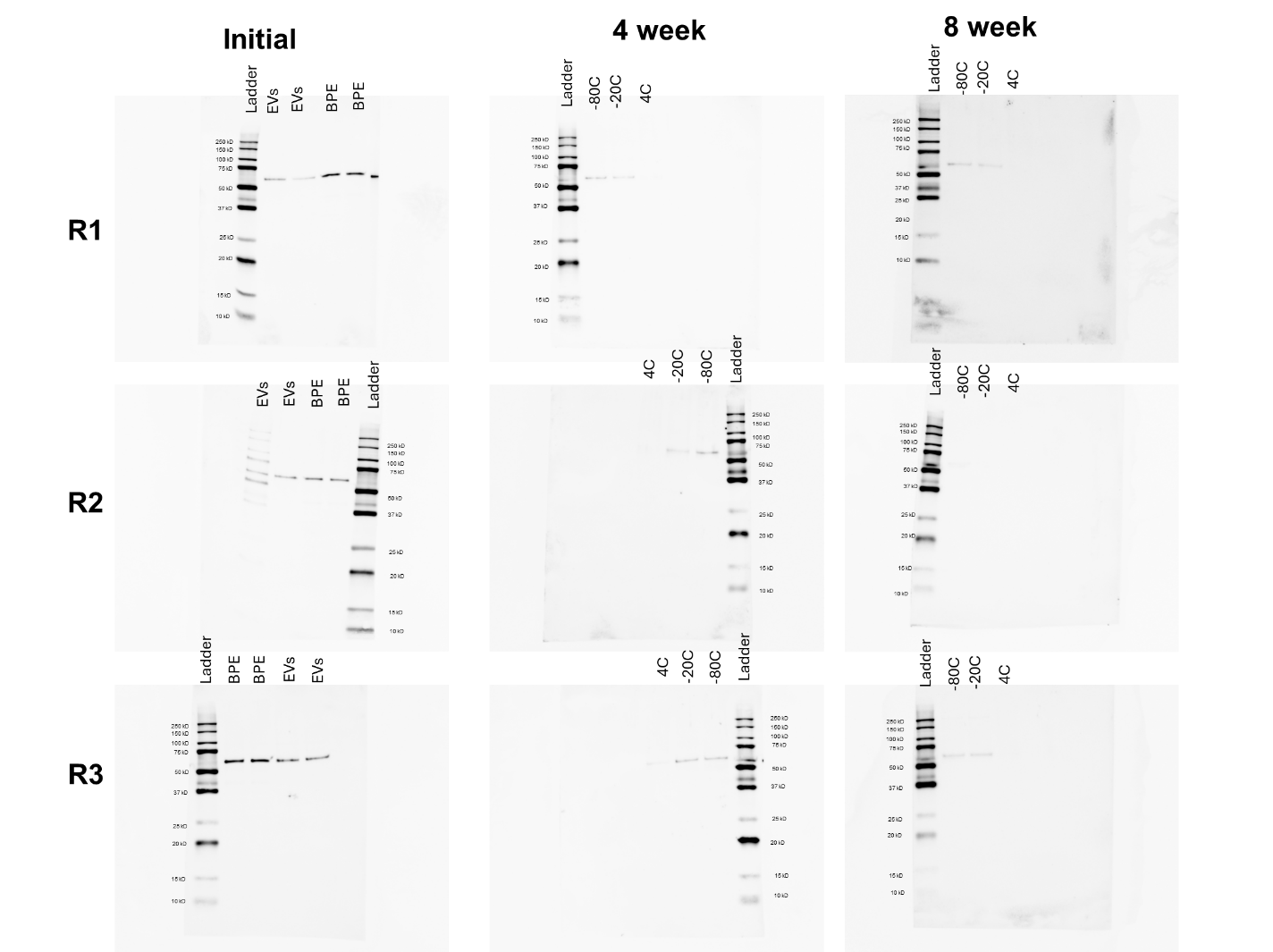


**Figure S4.** *B. longum* GroEL western blots at initial, 4-week, and 8-week timepoints. Initial images include both EVs and BPEs, while 4-week and 8-week timepoints include EVs after storage at 4 °C, -20 °C, or -80 °C.


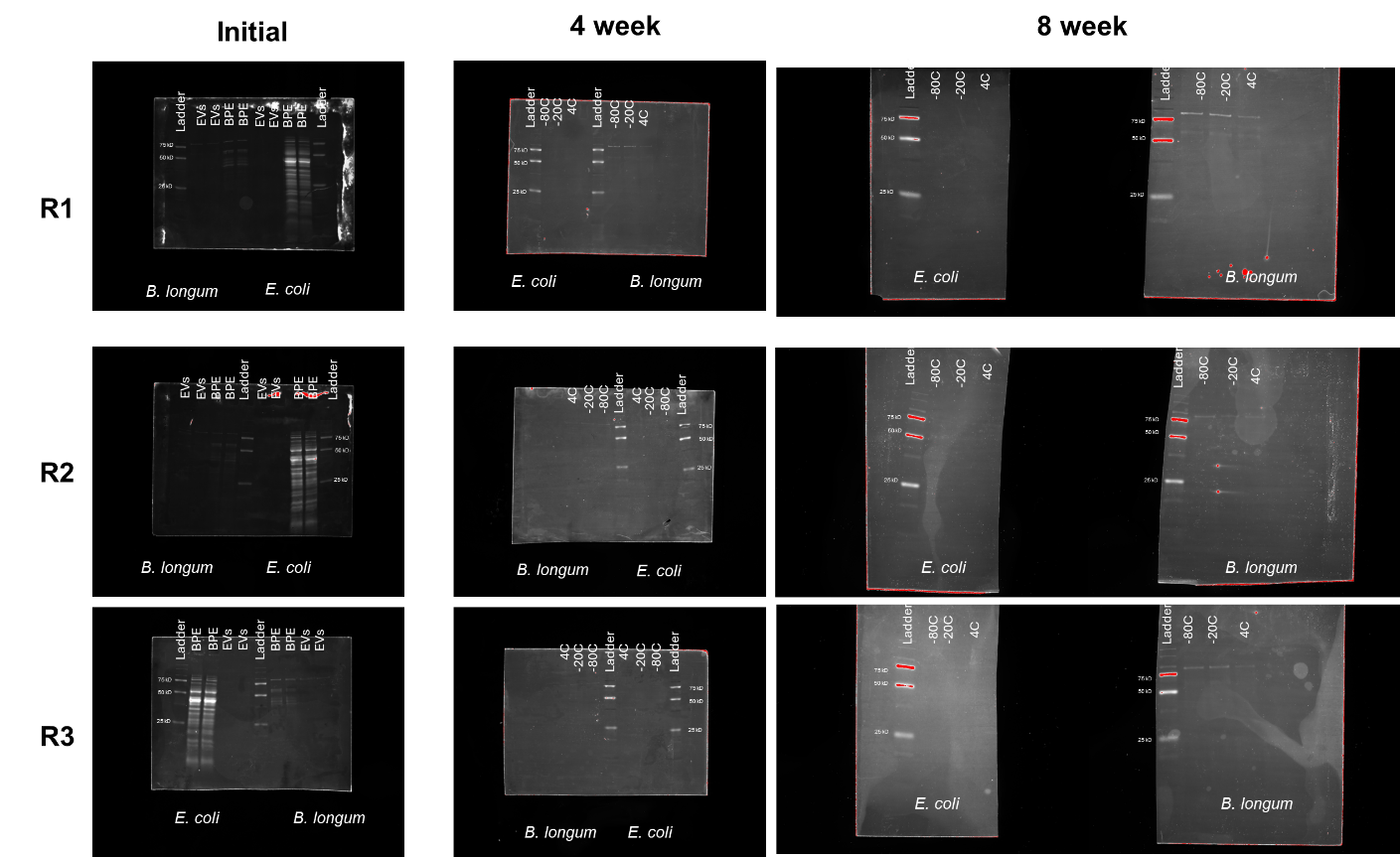


**Figure S5.** Total protein stain on *B. longum* and *E. coli* GroEL western blots at initial, 4-week, and 8-week timepoints. Initial images include both EVs and BPEs, while 4-week and 8-week timepoints include EVs after storage at 4 °C, -20 °C, or -80 °C.


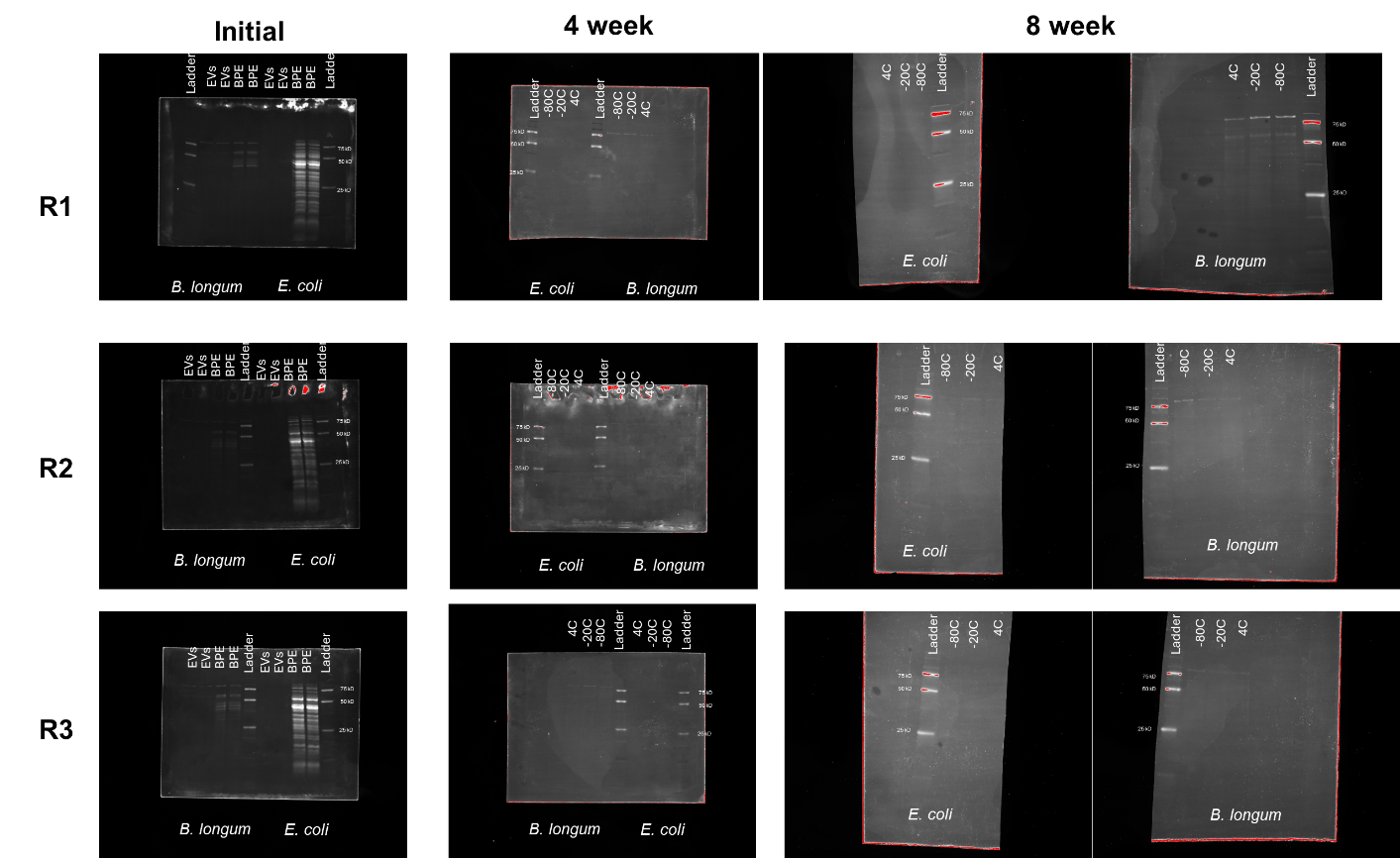


**Figure S6.** Total protein stain on *B. longum* RecA and *E. coli* flagellin western blots at initial, 4-week, and 8-week timepoints. Initial images include both EVs and BPEs, while 4-week and 8-week timepoints include EVs after storage at 4 °C, -20 °C, or -80 °C.


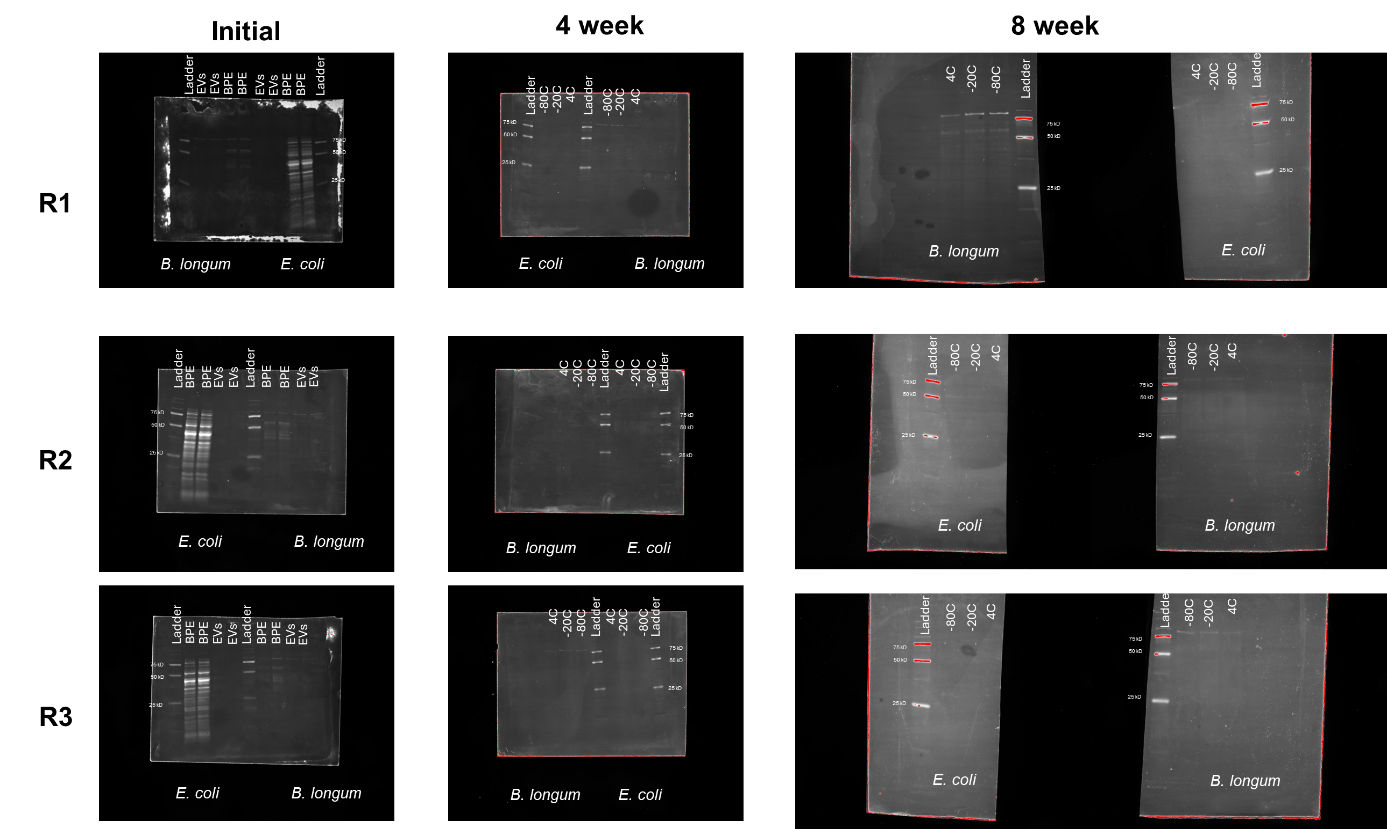


**Figure S7.** Total protein stain on *B. longum* LTA and *E. coli* LPS western blots at initial, 4-week, and 8-week timepoints. Initial images include both EVs and BPEs, while 4-week and 8-week timepoints include EVs after storage at 4 °C, -20 °C, or -80 °C.


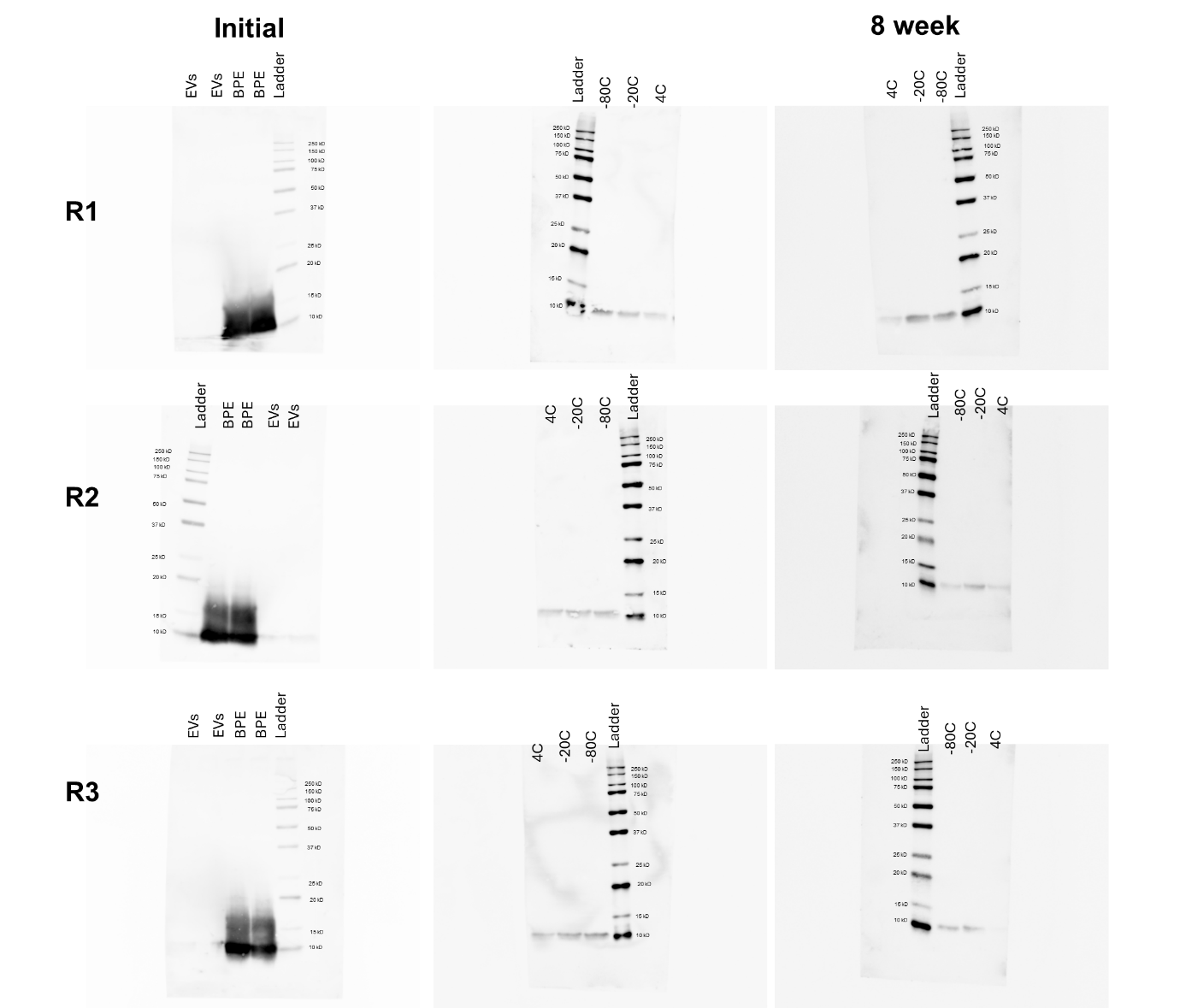


**Figure S8.** *E. coli* LPS western blots at initial, 4-week, and 8-week timepoints. Initial images include both EVs and BPEs, while 4-week and 8-week timepoints include EVs after storage at 4 °C, -20 °C, and -80 °C.


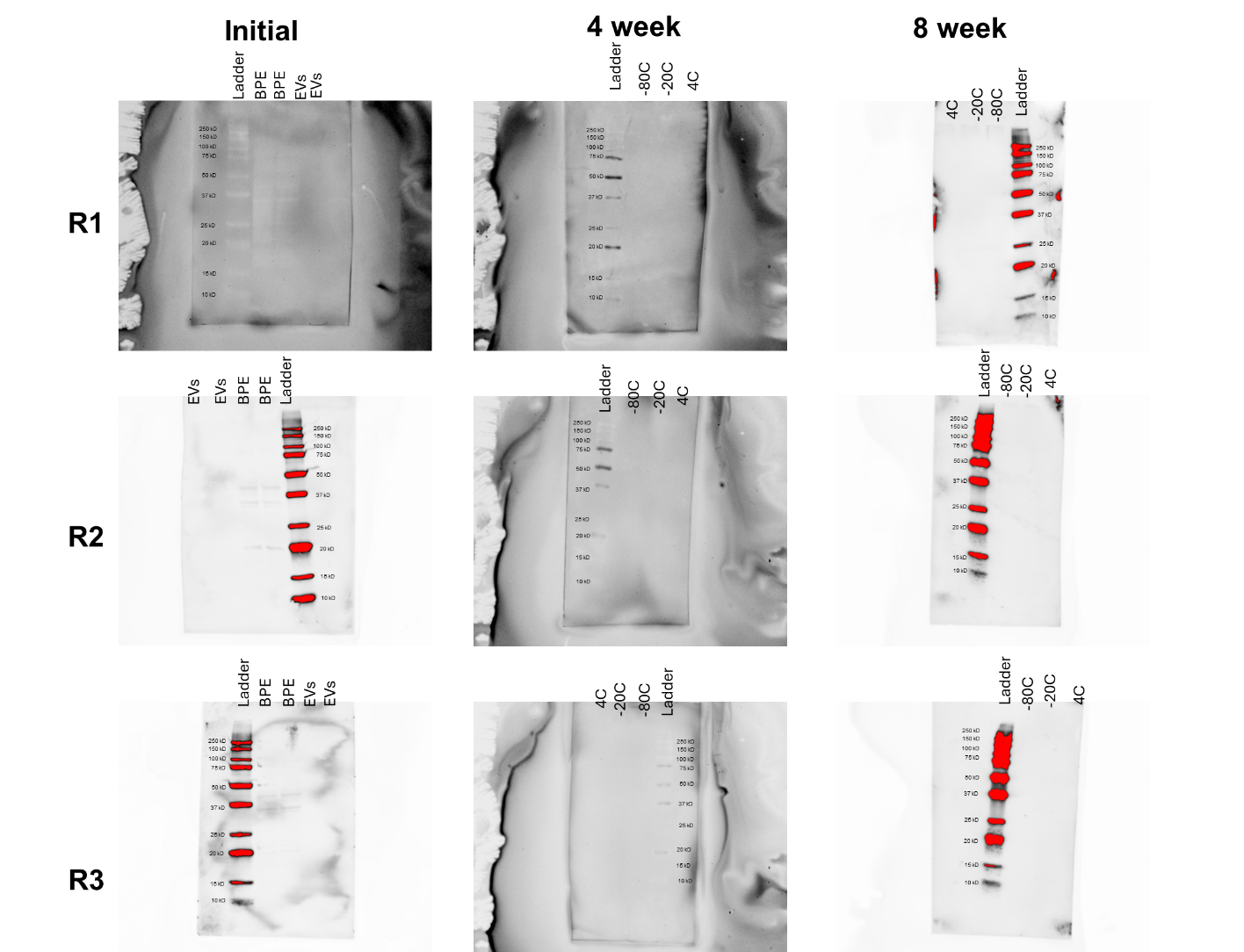


**Figure S9.** *E. coli* flagellin western blots at initial, 4-week, and 8-week timepoints. Initial images include both EVs and BPEs, while 4-week and 8-week timepoints include EVs after storage at 4 °C, -20 °C, and -80 °C. R3 was excluded from EV:BPE band ratio quantification due to high background signal and resulting imaging artifacts in the R3 initial timepoint blot.


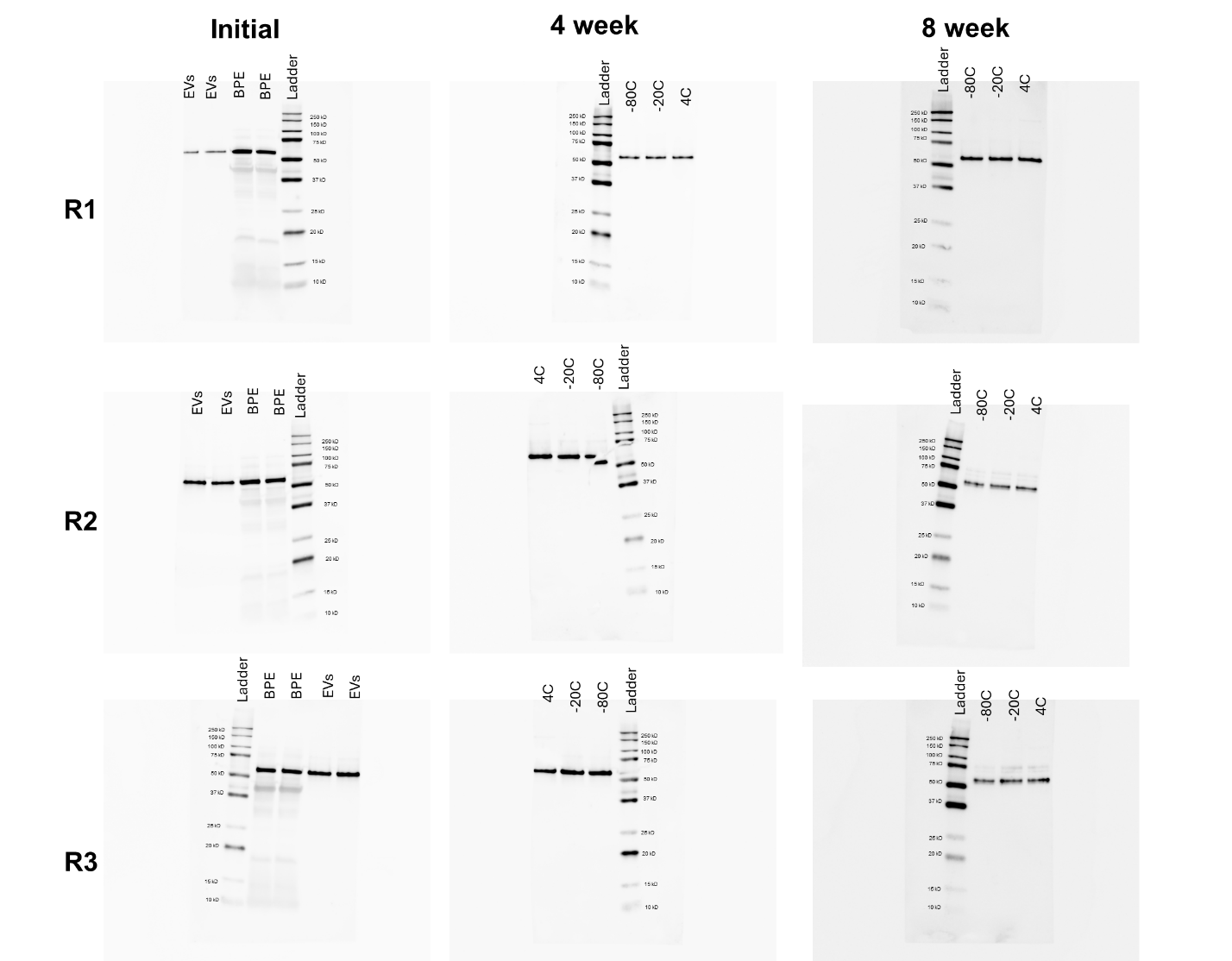


**Figure S10.** *E. coli* GroEL western blots at initial, 4-week, and 8-week timepoints. Initial images include both EVs and BPEs, while 4-week and 8-week timepoints include EVs after storage at 4 °C, -20 °C, or -80 °C.


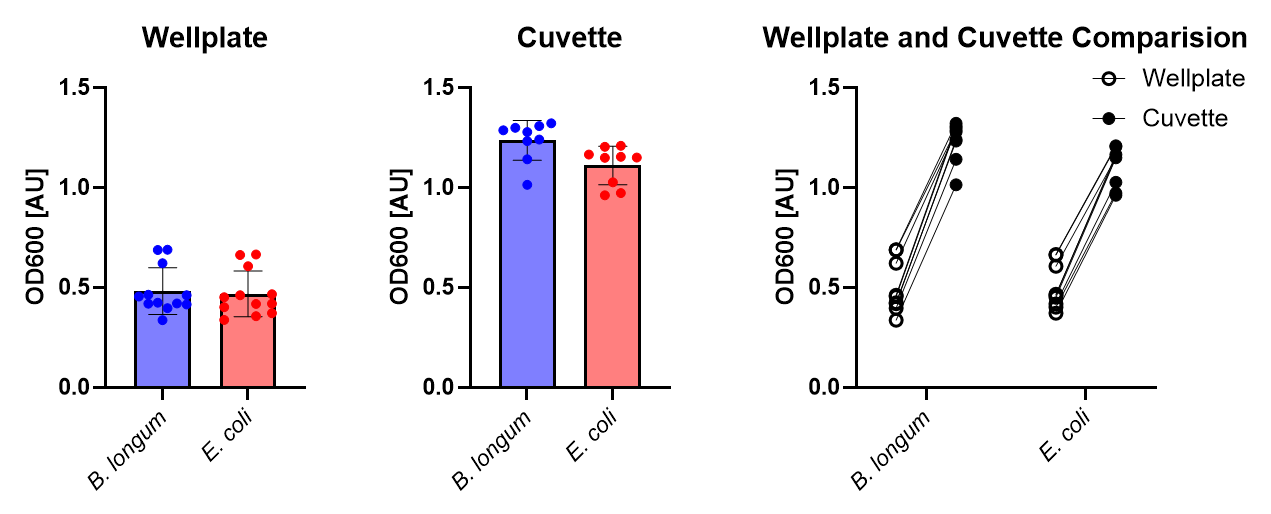


**Figure S11.** Wellplate versus cuvette optical density (OD600) measurement of *B. longum* and *E. coli* cultures.


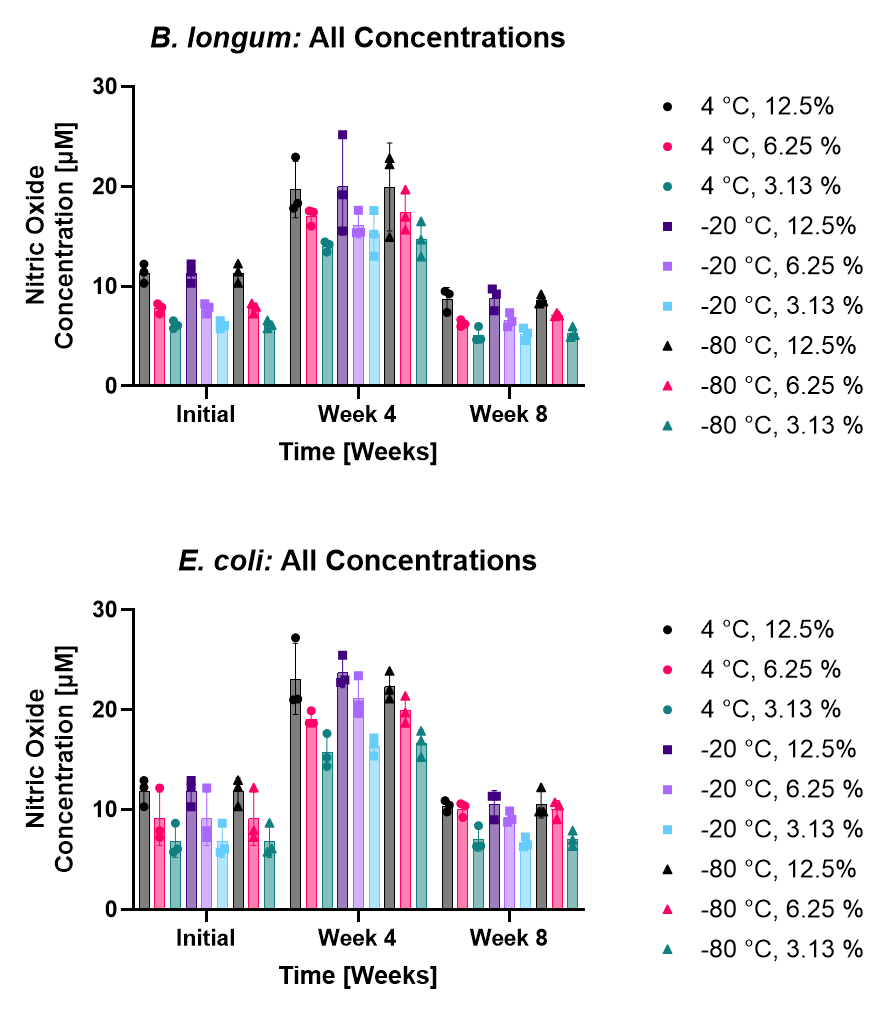


**Figure S12.** Dose response of RAW264.7 activation (measured by nitric oxide concentration in culture medium) to varying concentrations of bacterial EVs. Percentages represent final v/v concentrations.

**Figure S13.** RAW264.7 activation (measured by nitric oxide concentration in culture medium) by vehicle control.

**Figure S14.** *E. coli* OD600 values collected over a culture time of 30 hours. By 24 hours, the culture had departed from the logarithmic growth phase. Data represents average and standard deviation across n=3 replicates.

**Supplemental Methods**

***B. longum* Culture.** Reinforced Clostridial Medium was prepared by dissolving 38 g of the dehydrated media in 1 L of MilliQ water, which was then heated to boiling and sterilized by autoclave. Prepared medium was aliquoted in 10-mL aliquots and stored in an anaerobic chamber at 4-8 °C until use. For each replicate, an initial inoculation of a 10 mL tube of prepared medium was performed from frozen stock using the loop inoculation method. The bacteria was allowed to grow for three days under anaerobic conditions in an incubator at 37 °C without shaking, before a second inoculation was performed. For the second inoculation, for each replicate 1 mL of bacterial suspension from the initial tube was added to three fresh tubes containing 10 mL of medium each. The newly inoculated tubes (three per replicate) were incubated for a further three days under anaerobic conditions at 37 °C without shaking. The tubes used for each replicate were kept in a separate anaerobic chamber from tubes used for other replicates, and the inoculations for each replicate were staggered to avoid inoculating multiple replicates of the same bacterial strain on the same day. The three final tubes for each replicate were combined for use in EV isolation.

***E. coli* Culture.** The medium was prepared by dissolving 15 g of dehydrated medium in 500 mL MilliQ water followed by heating while stirring on a stir plate until boiled for 1 minute. Medium was sterilized by autoclave and stored at 4-8 °C until use. For each replicate, an initial inoculation of a 10 mL tube of prepared medium was performed from frozen stock using the loop inoculation method. The bacteria was allowed to grow for a 24 hour period in the incubator at 37 °C with shaking at 100 rpm, before a second inoculation was performed. For the second inoculation, for each replicate 1 mL of the bacteria from the initial tube was added to each of three fresh tubes containing 10 mL of medium each. The newly inoculated tubes (three per replicate) were incubated for an additional 24 hours at 37°C with shaking at 100 rpm. The inoculations for each replicate were staggered to avoid inoculating multiple replicates of the same bacterial strain on the same day. The three final tubes for each replicate were combined for use in EV isolation.

**EV Isolation and Storage.** Prior to EV isolation, bacteria were removed from the incubator and 100 µL was taken from each tube (3 per replicate) for reading OD600, with the remaining volume used for EV isolation. After EV isolation and collection of initial measurements from the EVs, 100 µL aliquots were prepared for storage and divided evenly among the three temperature conditions. For EVs used in macrophage activation experiments, EVs were eluted in a laminar flow biosafety cabinet using EV elution buffer that was handled with aseptic technique. These EVs were stored in sterile 0.65-mL plastic centrifuge tubes.

**Nanoparticle Tracking Analysis.** All NTA protocols included data collection at 11 positions per measurement, with 3 measurements taken per sample. Data from certain positions were automatically excluded by the software for quality control purposes, such as if too few or too many particles were detected in the field of view. The results from the three measurements of each sample were averaged to obtain the value for that sample. Wherever possible, measurements with 8 or more usable positions were used in our analyses.

**Gel Electrophoresis.** For silver staining, gels were fixed for 15 minutes in a solution of 50% (v/v) methanol, 30% (v/v) water, 10% (v/v) acetic acid, 10% (v/v) Fixative Enhancer Solution from kit manufacturer. The gels were then rinsed twice in 100 mL of DI water for 20 minutes. The gels were submerged in staining solution, with gentle agitation, for 10 minutes. The reaction was then stopped with 5% acetic acid. ChemiDoc Imaging System, software version 3.0.1.14, firmware version 1.16 r. An exposure time of 0.2 seconds was used to capture each image.

**Western Blot Total protein Stain.** The membranes were first floated face down onto solution of 7% acetic acid, 10% methanol for 15 minutes. They were then washed in four changes of DI water for 5 minutes and then floated onto SYPRO Ruby stain for 15 minutes. The excess dye was removed with a final DI water wash for 1 minute. Images were captured with exposure times of 0.01 and 0.1 seconds.

**Western Blot Antibody-Based Staining.** Primary antibodies were diluted to the following concentrations: Anti-GroEL (1:20,000), Anti-Flagellin (1:500), Anti-Rad51 (1:500), Anti-LPS (1:1000), and Anti-LTA (1:63). The membranes probed with the GroEL antibody solutions were incubated for 1 hour at room temperature, while the rest of the membranes were incubated for 2 hours. Secondary antibodies were diluted 1:10000.
